# Supplementary material for: Correlative molecular-to-mesoscale evolution in conjugated polymers for intrinsically stretchable organic photovoltaics
Source: Nat Commun. 2026 Feb 20;17:2980. doi: 10.1038/s41467-025-68265-4 (PMC13035893; doi:10.1038/s41467-025-68265-4)
Supplement: Supplementary file 1 — Supplementary Information [file 41467_2025_68265_MOESM1_ESM.pdf]

Supplementary Information

Correlative molecular-to-mesoscale evolution in conjugated polymers for intrinsically stretchable organic photovoltaics

Wenkai Zhong,[1,2,*] Guillaume Freychet,[3,4] Gregory M. Su,[5,6] Siyi Wang,[1] Xuanang Luo,[1] Xinrui Liu,[1] Wenyu Yang,[1] Lei Yu,[1] Xuefei Wu,[6] Yulong Li,[1] Thomas J. Ferron,[5] Thomas P. Russell,[6,7] Lei Ying,[1] Fei Huang,[1] Yongming Zhang,[2] Cheng Wang,[5,*] Feng Liu[2,*]

[1] Institute of Polymer Optoelectronic Materials and Devices, Guangdong Basic Research Center of Excellence for Energy and Information Polymer Materials, State Key Laboratory of Luminescent Materials and Devices, South China University of Technology, Guangzhou 510640, China
E-mail: wkzhong@scut.edu.cn

[2] Frontiers Science Center for Transformative Molecules, Center of Hydrogen Science, School of Chemistry and Chemical Engineering, Shanghai Jiao Tong University, Shanghai 200240, China
E-mail: fengliu82@sjtu.edu.cn

[3] NSLS-II, Brookhaven National Laboratory, Upton, NY 11973, United States

[4] Univ. Grenoble Alpes, CEA, Leti, F-38000 Grenoble, France

[5] Advanced Light Source, Lawrence Berkeley National Laboratory, Berkeley, CA 94720, United States
E-mail: cwang2@lbl.gov

[6] Materials Sciences Division, Lawrence Berkeley National Laboratory, Berkeley, CA 94720, United States

[7] Polymer Science and Engineering Department, University of Massachusetts, Amherst, Massachusetts 01003, United States

Supplementary Figures

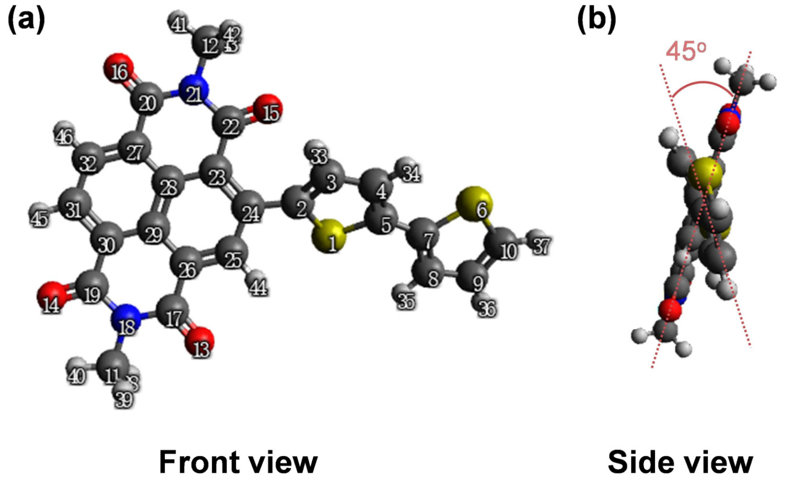

Supplementary Figure 1. Molecular model of a single P(NDI2OD-T2) repeat unit calculated with DFT method with B3LYP/6-31G(d) basis: (a) front view; (b) side view. The alkyl side chains were replaced with methyl groups to simplify the calculations.

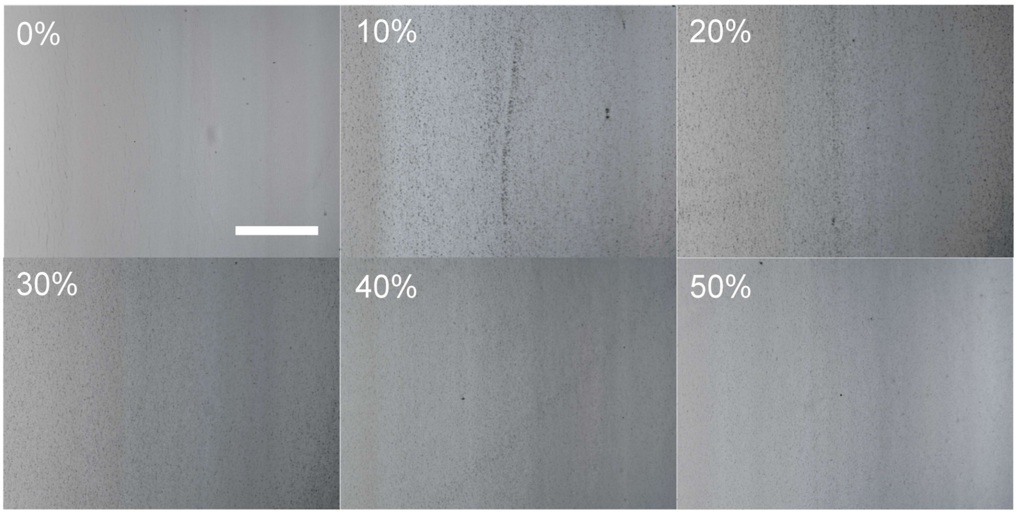

Supplementary Figure 2. Optical images of P(NDI2OD-T2) film-on-elastomer sample with various strains; no clear crack is observed when the film is stretched with 50% strain, indicating that crack onset strain is larger than 50%. The scale bar represents a length of 500 μm.

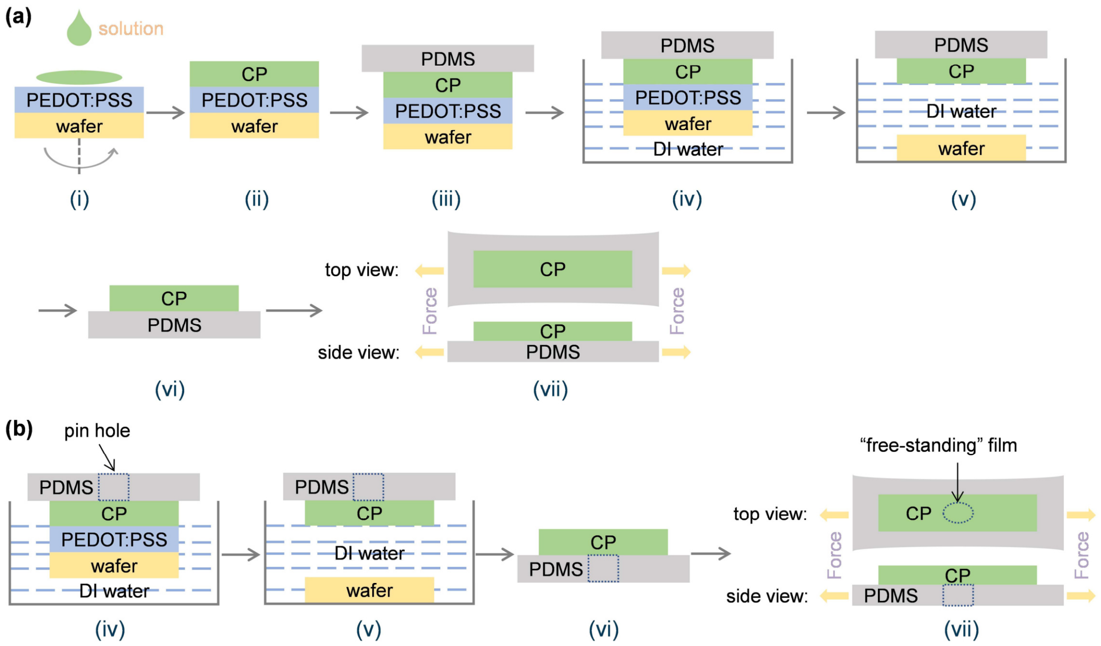

Supplementary Figure 3. A fast and universal transfer method for conjugated polymer (CP) thin films onto a stretchable substrate: (a) transfer procedure for film-on-elastomer sample; (b) transfer procedure for free-standing sample. The film-transfer procedure: (i) CP solution is spin-coated on a PEDOT:PSS/wafer substrate; (ii) post-treatment is applied to the film sample; (iii) the film surface is covered with a PDMS membrane; (iv) the PDMS/CP/PEDOT:PSS/wafer sample is transferred on water; (v) the CP/PDMS is separated and floats on water as PEDOT:PSS dissolves in water; (vi) the CP/PDMS is moved to a glass slide and dried in air; (vii) uniaxial tensile force is applied to the PDMS, providing strain to the CP thin film.

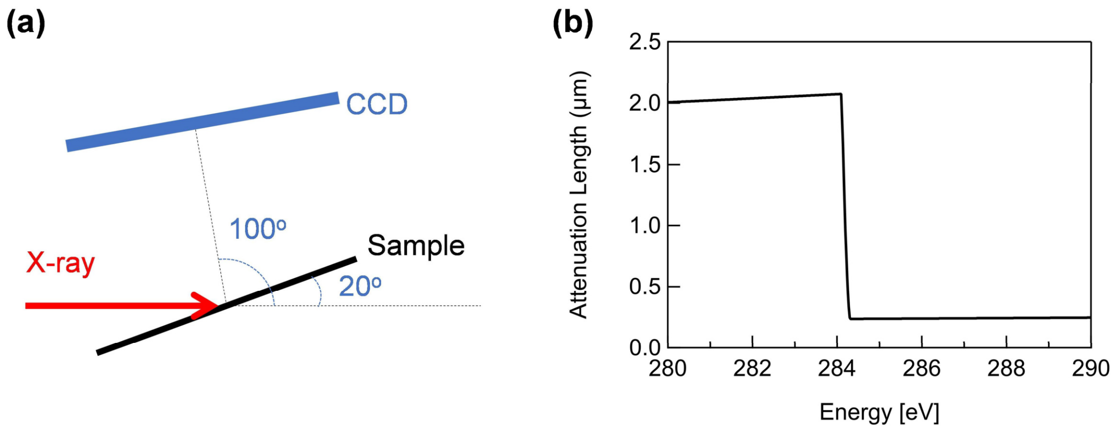

Supplementary Figure 4. (a) Front-view of the NEXAFS experimental geometry. The thin film surface was inclined at a 20° angle with respect to the beam path. The emitted photons from the thin film were detected by a 2D detector positioned at a 100° tilt relative to the beam path. (b) The attenuation length of X-ray into a 200-nm-thick P(NDI2OD-T2) thin film under the energy range of 280-290 eV; the mass density of P(NDI2OD-T2) was assumed as 1.0 g cm-3 and the incident angle (the fluorescent photons from the bulk to surface) was set at 90°. It is seen that the attenuation length is higher than 200 nm through the X-ray energy range of 280-290 eV. This means that self-absorption is negligible for TFY collection from a 200-nm-thick P(NDI2OD-T2) thin film during stretching.

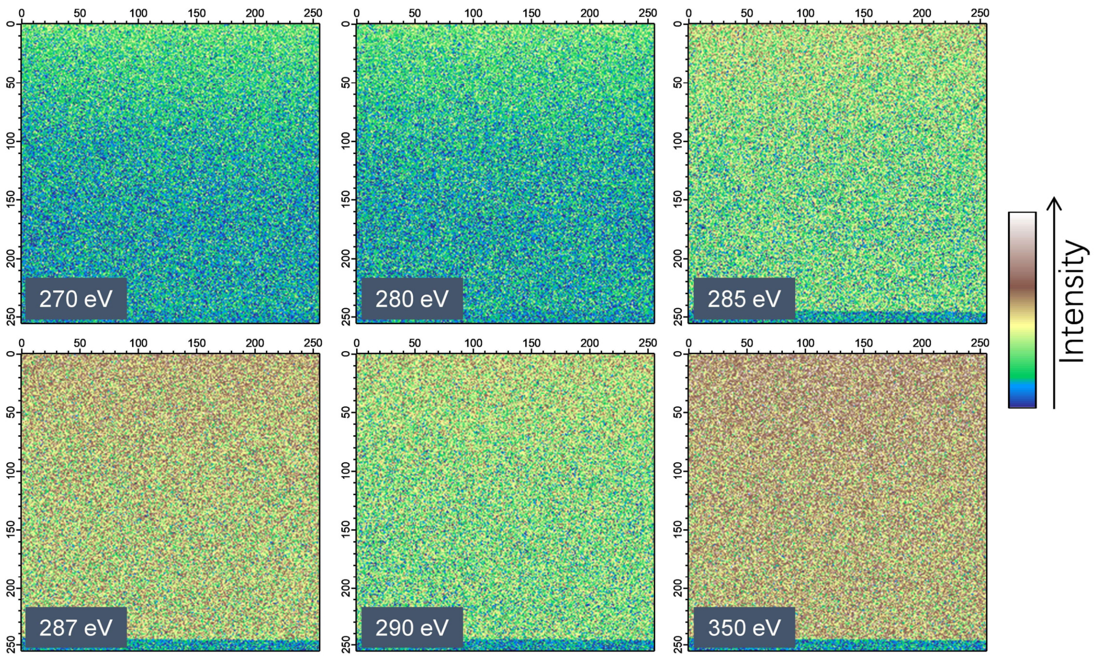

Supplementary Figure 5. Representative CCD images captured at various energies during TFY NEXAFS spectroscopy measurement of a P(NDI2OD-T2) thin film. The exposure time at each image is 0.5 s. The pixel binning was set as 8 × 8 to speed up the data acquisition.

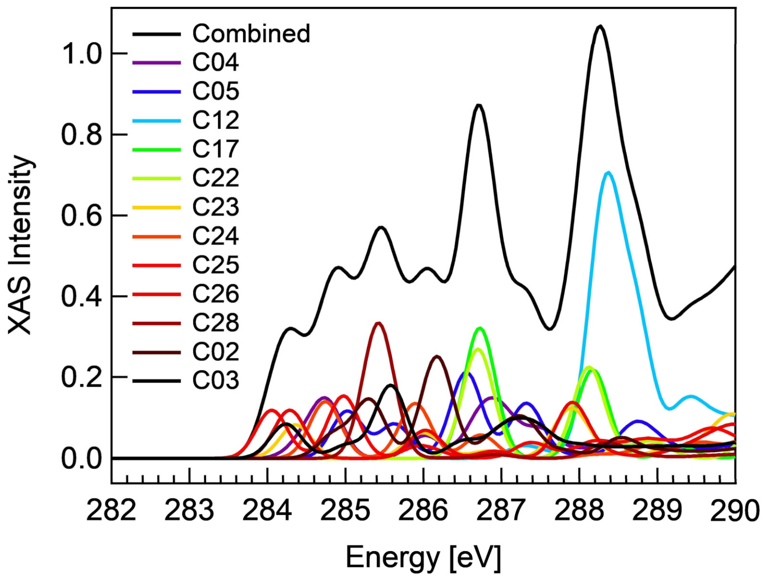

Supplementary Figure 6. Simulated NEXAFS spectra of a single P(NDI2OD-T2) repeat unit. The numbers in the legend are corresponding to the carbon labels indicated in the molecular model shown in Fig S1. The XAS intensity of the combined curve is scaled for better comparison.

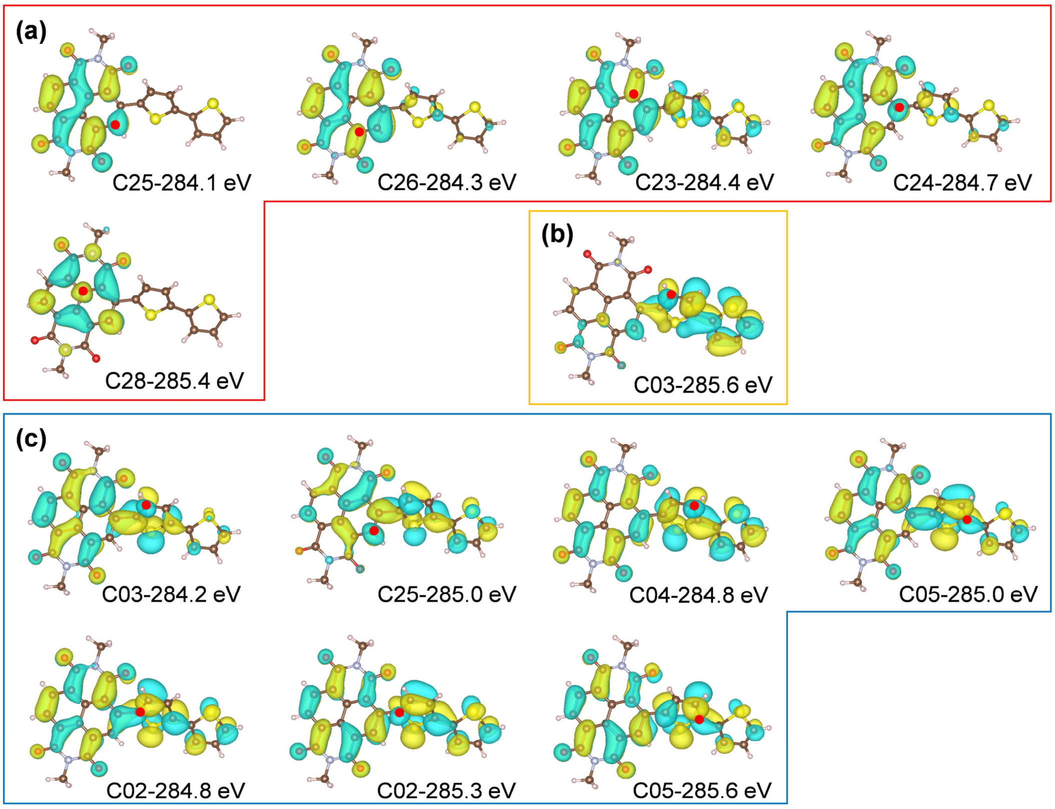

Supplementary Figure 7. Categories of 1s→π* transitions according to the density distributions of electronic final-state orbitals (a) localized to the NDI unit, (b) localized to the BT unit, and (c) delocalized over NDI and BT units. The inset labels are the C atoms consistent with those of Supplementary Figure 6 and corresponding energies.

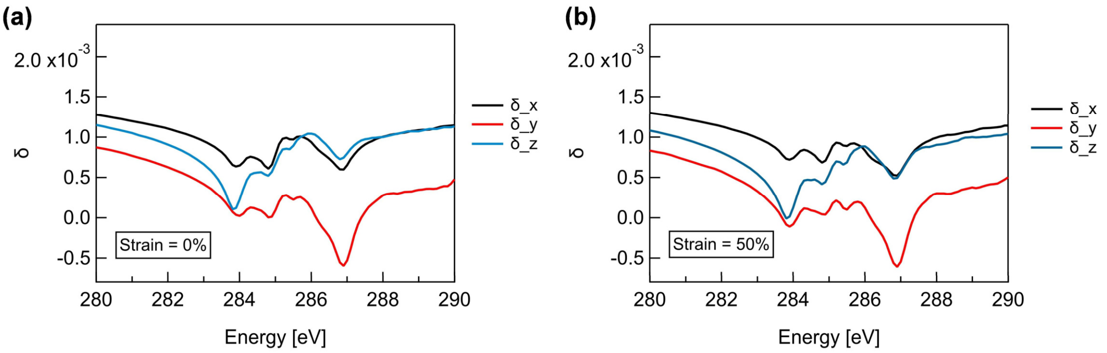

Supplementary Figure 8. Directional refractive indices (δ) for P(NDI2OD-T2) thin films under (a) pre-stretched and (b) 50% strain conditions.

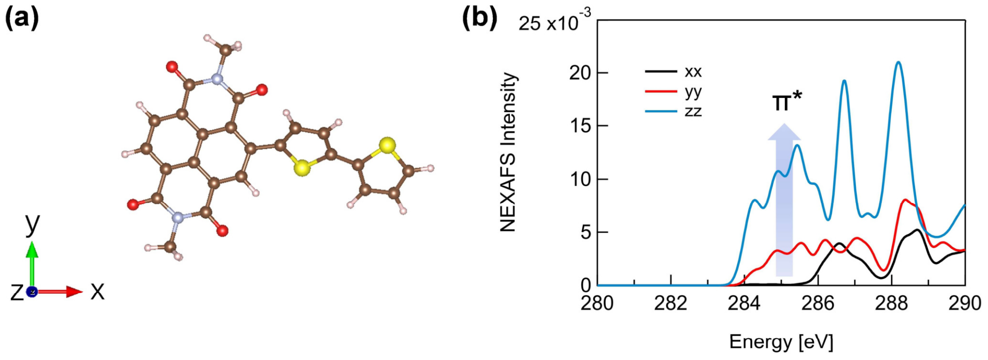

Supplementary Figure 9. (a) Illustration of the x, y, and z directions of the P(NDI2OD-T2) molecular model for NEXAFS simulations by XCH method. (b) The resulted x, y, and z components of the resulted NEXAFS spectra. The C=C 1s→π* transitions (283.5-286.0 eV) have the intensity follows a trend of $I_z > I_y > I_x$, which suggest that the NEXAFS intensity is minimized when the polymer backbone is aligned in the x axis.

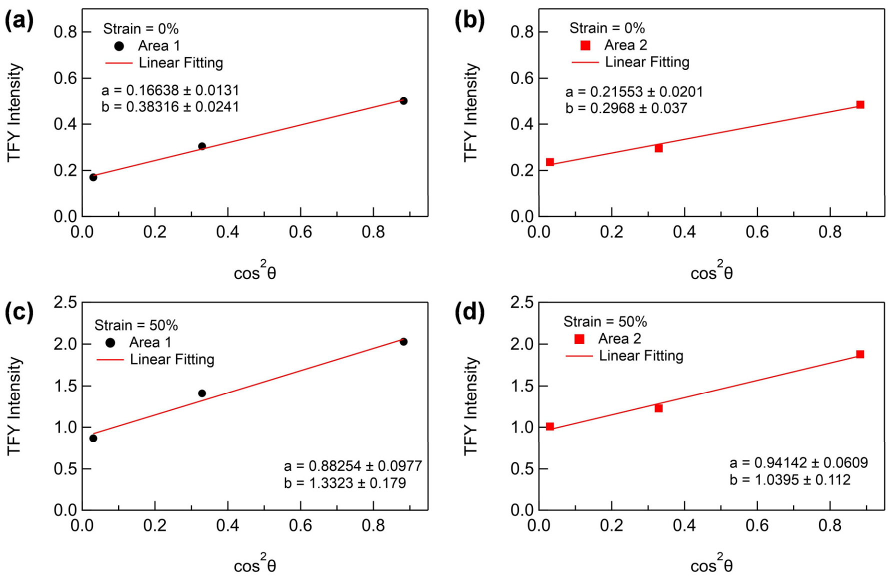

Supplementary Figure 10. Linear-fitting results of angle-dependent TFY NEXAFS spectroscopy data of P(NDI2OD-T2) thin films: (a) Area 1 at 0% strain; (b) Area 2 at 0% strain; (c) Area 1 at 50% strain; and (d) Area 2 at 50% strain. Parameters a and b represent the resultant intercept and slope, respectively. The area under the curve was directly calculated from a fixed energy window.

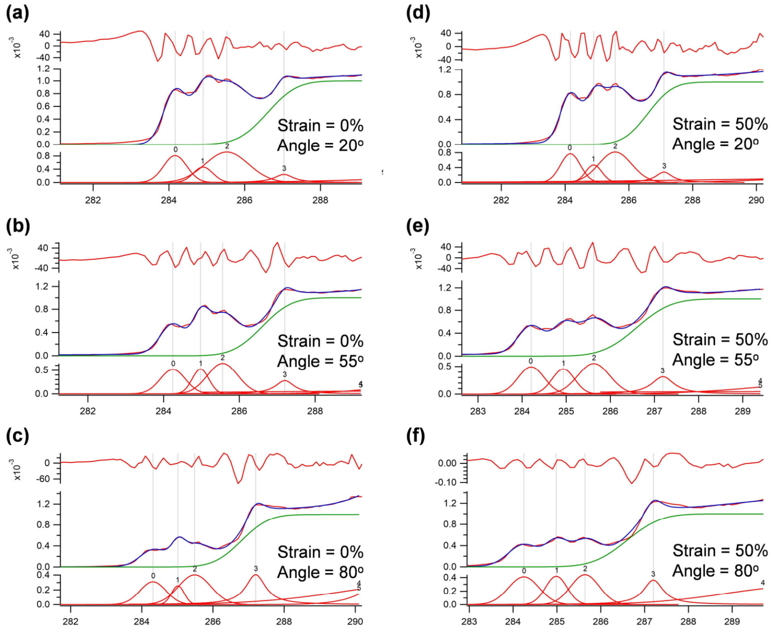

Supplementary Figure 11. Gaussian multi-peak fitting of the π* region in angle-dependent NEXAFS spectra of P(NDI2OD-T2) thin films: (a-c) under 0% strain; (d-f) under 50% strain.

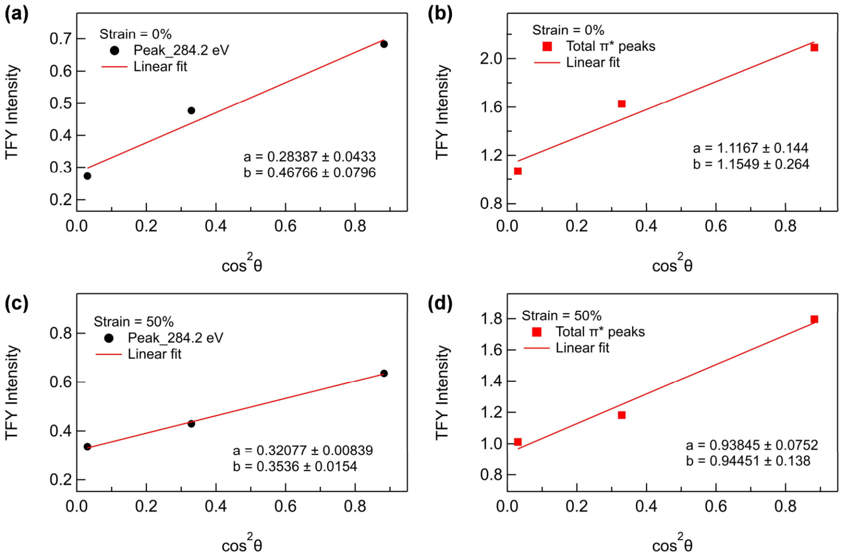

Supplementary Figure 12. Linear fitting of peak area of angle-dependent TFY NEXAFS spectra, obtained from Gaussian multi-peak fitting of P(NDI2OD-T2) thin films: (a) Peak at 284.2 eV under 0% strain; (b) Total π* peaks under 0% strain; (c) Peak at 284.2 eV

under 50% strain; and (d) Total π* peaks under 50% strain. Parameters a and b represent the resultant intercept and slope, respectively.

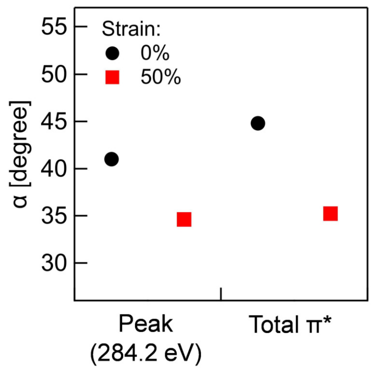

Supplementary Figure 13. Fitted tilt angles (α) derived from Gaussian multi-peak fitting for the peak at 284.2 eV and the total π* region. The extracted α values for the 50% strain thin film are 34.6° and 35.2°, representing decreases of 6.4° and 9.5°, respectively, compared to the pre-stretched film. These results are consistent with those obtained from direct integration (Supplementary Figure 10), confirming the same trend in tilt angle reduction under strain, with only minor numerical differences.

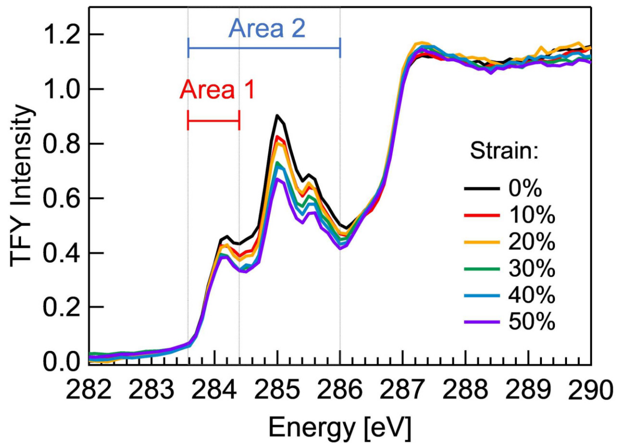

Supplementary Figure 14. TFY spectra of a P(NDI2OD-T2) thin film under various strains. The X-ray is horizontally polarized, as well as parallel to the stretch direction of the thin film.

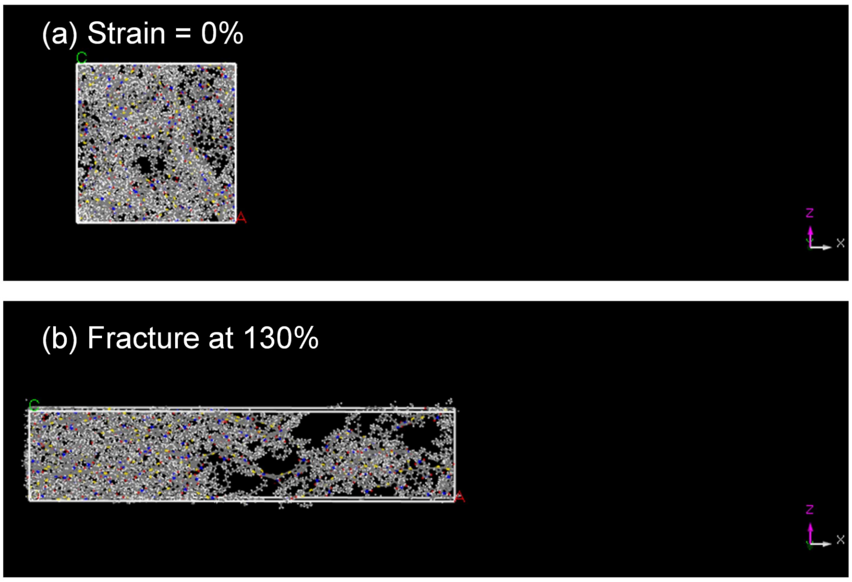

Supplementary Figure 15. Snapshots of P(NDI2OD-T2) chains after stretched to fracture as simulated by molecular dynamics.

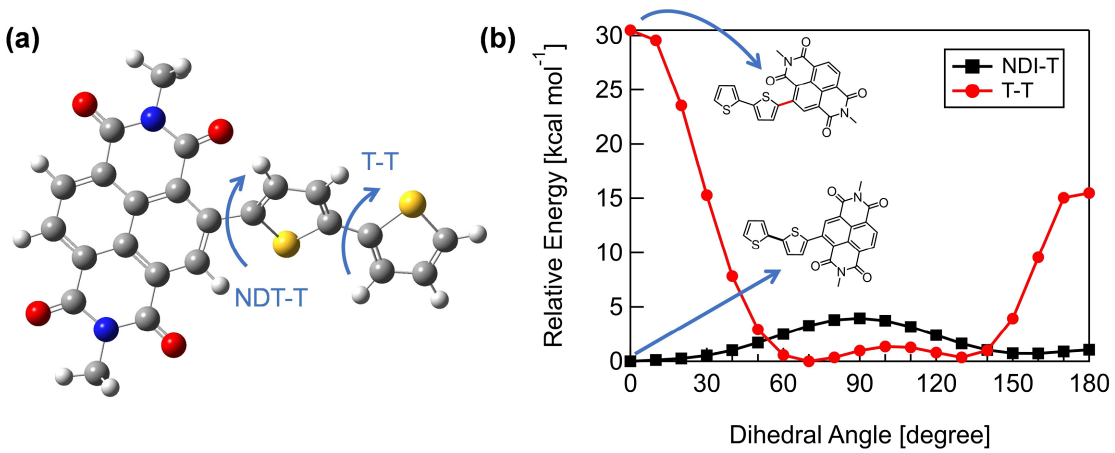

Supplementary Figure 16. (a) The molecular model for PES scan; (b) PES scan of the molecular model, where the insets represent the intrachain conformation when dihedral angle is set to zero.

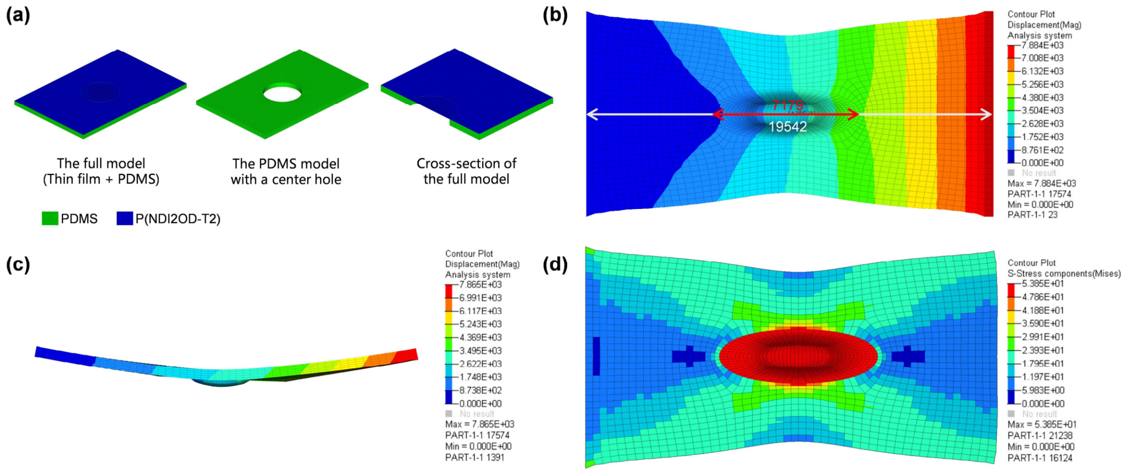

Supplementary Figure 17. Finite element analysis (FEA): (a) Simulated models of the P(NDI2OD-T2) thin film on a PDMS substrate with a central circular opening. (b) Simulated strain map, showing that the overall strain applied to the PDMS substrate (30%) leads to local strain amplification (79%) near the hole edge. The PDMS substrate and central hole have initial lengths of 15 cm and 0.4 cm, respectively. The inset numbers are in units of μm. (c) Side-view strain map, showing significant macroscopic bending under the perfect bonding assumption between the PDMS and P(NDI2OD-T2) film. (d) Simulated stress map, showing the stress concentration at the central hole, where the central 0.2 mm region of the unsupported film exhibits a nearly uniform uniaxial strain.

Supplementary Note 1: FEA simulations of the samples for TReXS and RSoXS were performed using Abaqus. The PDMS substrate was modeled as a rectangular block with dimensions of 10 cm × 15 cm × 0.5 cm, containing a circular opening of 4 mm in diameter at the center. A thin P(NDI2OD-T2) film (thickness: 0.2 μm) was conformally placed on the top surface of the PDMS, fully covering the hole region. A perfect bonding condition (no slip) was assumed between the P(NDI2OD-T2) film and PDMS, and the two layers were meshed using shared nodes. To simplify the computation while retaining the strain-distribution trend, both materials were described by linear elastic constitutive relations. The elastic moduli were set as 1.74 MPa for PDMS and 63.72 MPa for P(NDI2OD-T2). A static step analysis was used, where one side of the model was fully fixed while a 30% tensile strain was applied along the x-axis to the opposite side.

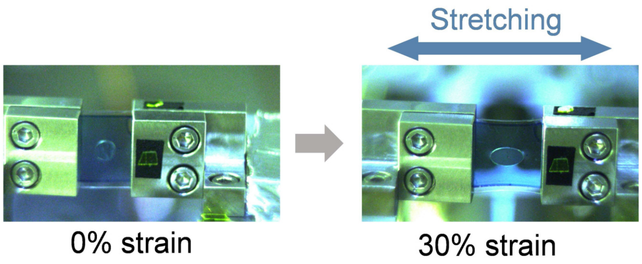

Supplementary Figure 18. The photos showing a P(NDI2OD-T2) thin film with 0% and 30% strain on the tensile tester. Such photos indicate no macroscopic bending occurs in practice, which reduces strain amplification and brings the actual film strain closer to the applied nominal value.

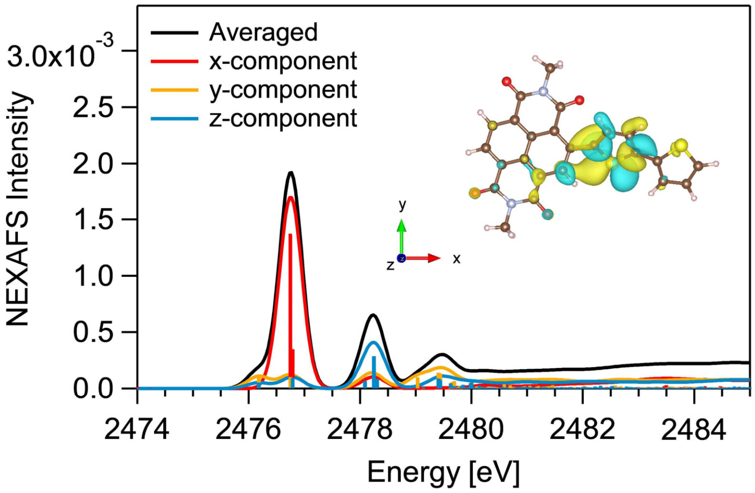

Supplementary Figure 19. Simulated S K-edge NEXAFS of P(NDI2OD-T2) and the corresponding x-, y-, and z-components. The x-component, which is in line with the axis of the conjugated backbone, shows much higher intensity than those of y- and z-components. Thus, the TDM of S 1s→σ* transition is roughly parallel to the conjugated backbone.

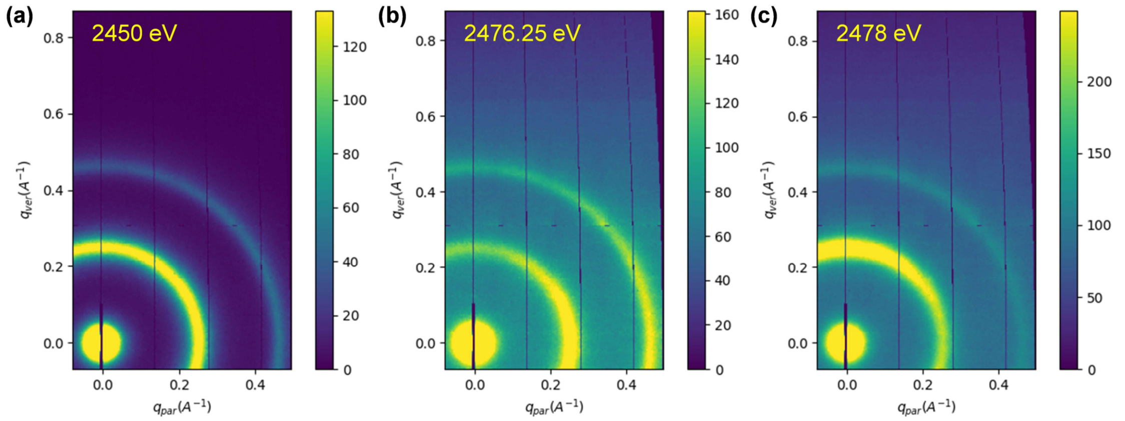

Supplementary Figure 20. TReXS patterns of an as-cast P(NDI2OD-T2) thin film captured under (a) pre-edge (2450 eV) and on-edge energies of (b) 2476.25 eV and (c) 2478 eV.

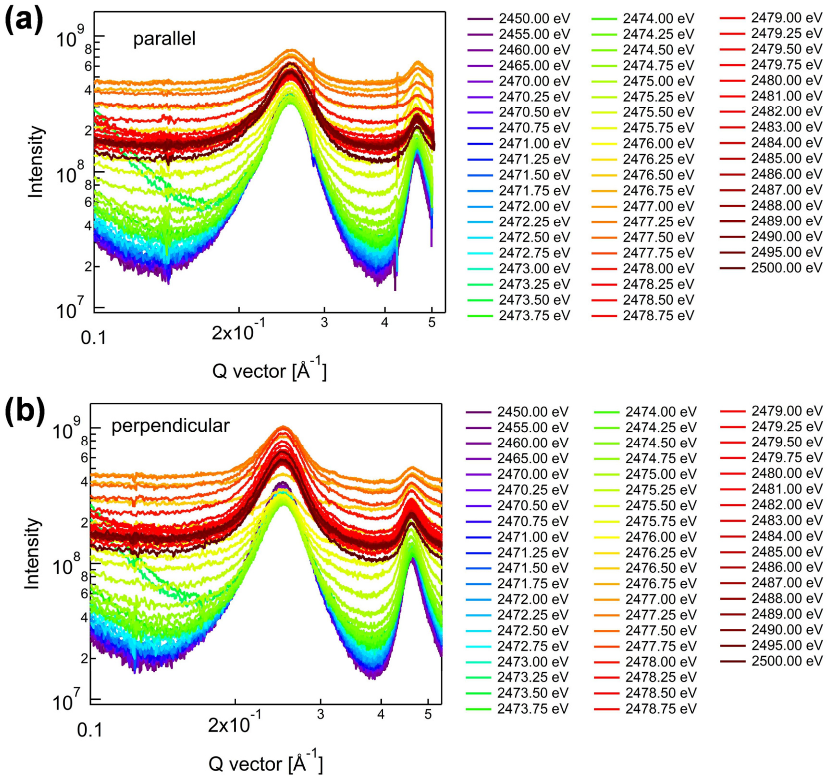

Supplementary Figure 21. Energy-dependent TReXS averaged *I-q* curves in the (a) parallel and (b) perpendicular directions.

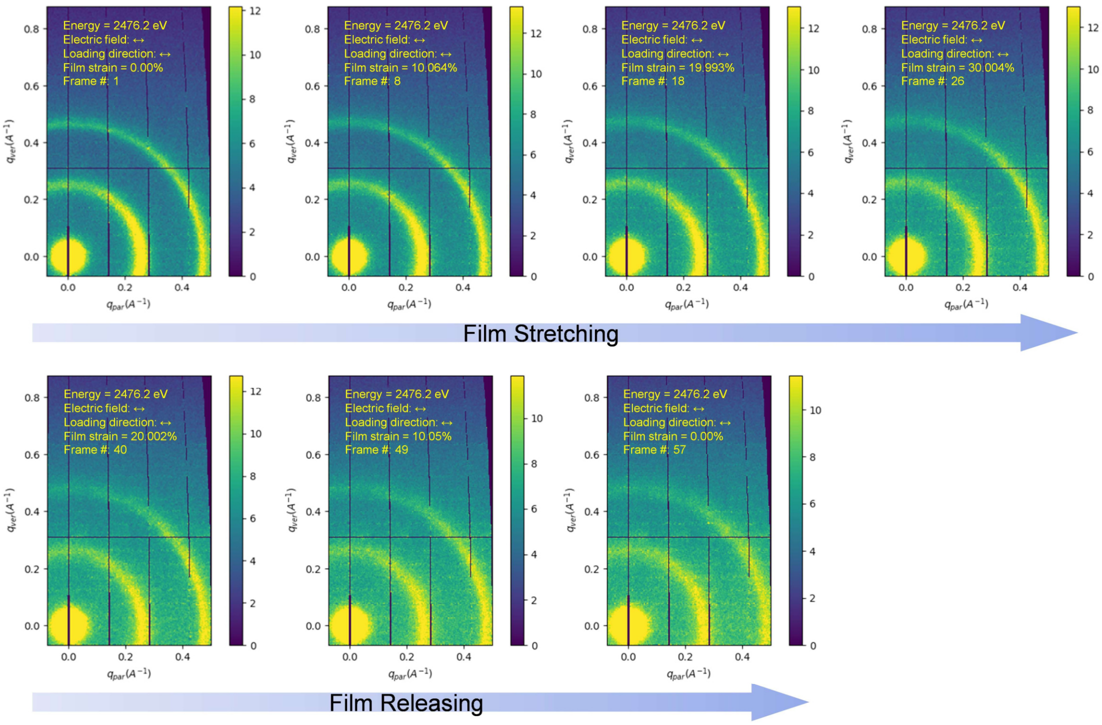

Supplementary Figure 22. Representative scattering patterns captured at 2476.2 eV during TReXS measurement for P(NDI2OD-T2) thin film upon tensile testing.

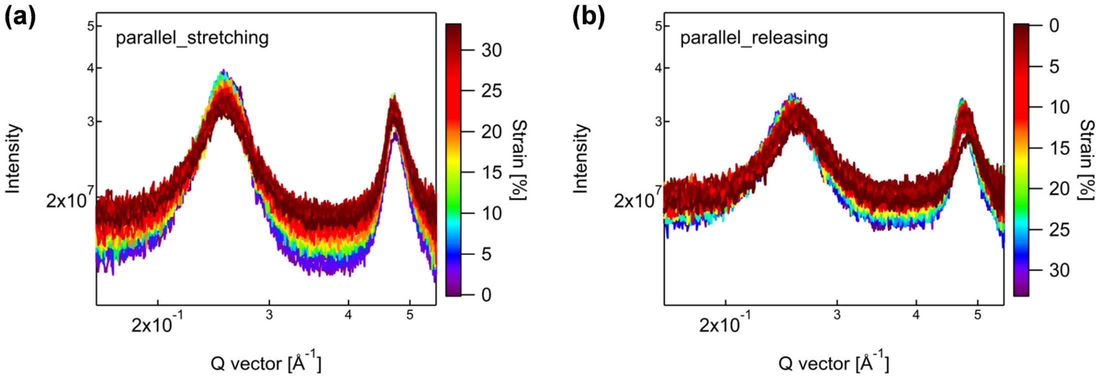

Supplementary Figure 23. Summary of TReXS *I*-*q* curves averaged in the parallel direction during the P(NDI2OD-T2) film (a) stretching and (b) releasing processes.

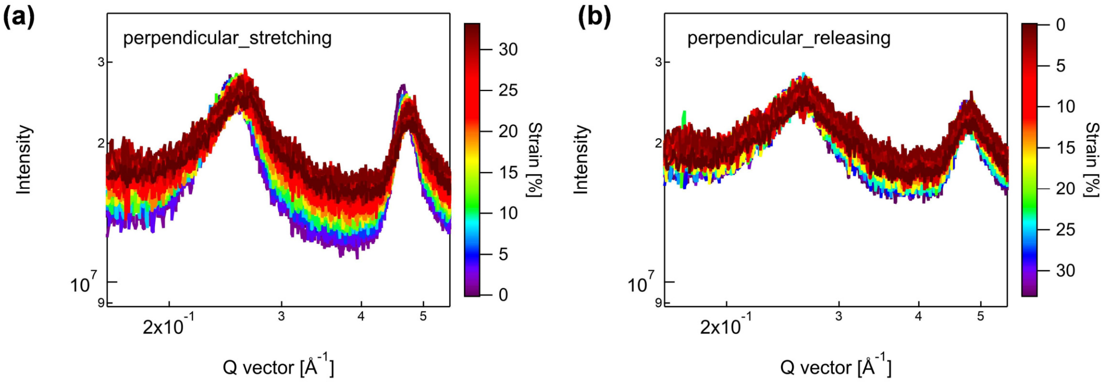

Supplementary Figure 24. Summary of TReXS *I*-*q* curves averaged in the perpendicular direction during the P(NDI2OD-T2) film (a) stretching and (b) releasing processes.

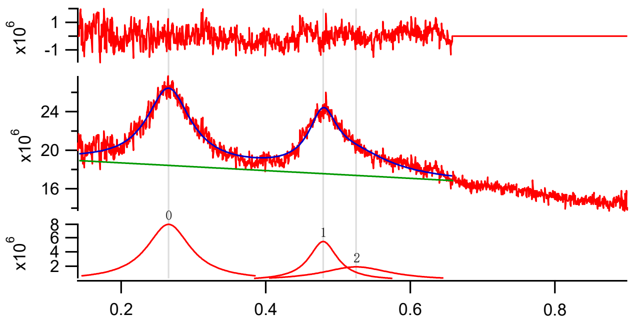

Supplementary Figure 25. Fitting example of the TReXS averaged curves for the extraction of peak intensities, where peak 0, 1, and 2 represent (100), (001), and (200) reflections, respectively.

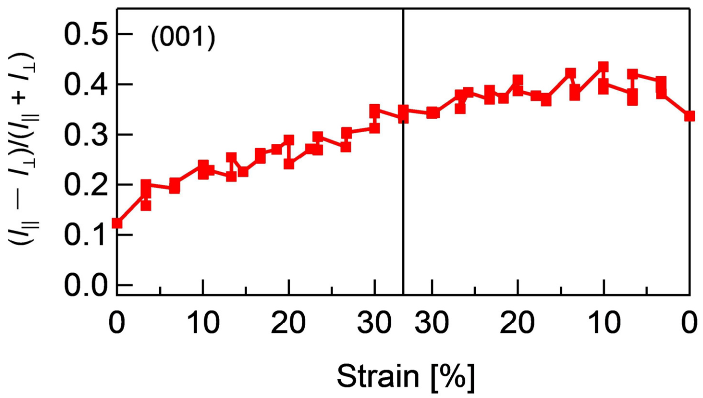

Supplementary Figure 26. Developments of anisotropic ratio of the P(DI2OD-T2) thin film during tensile testing.

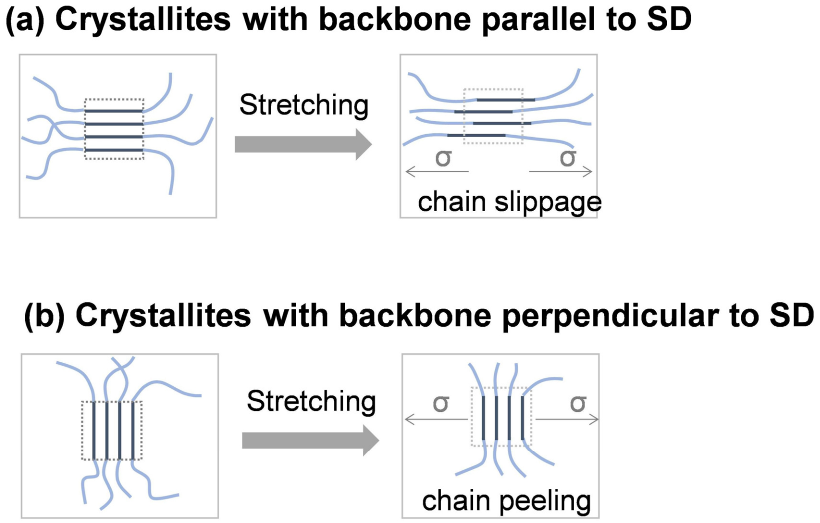

Supplementary Figure 27. Schematic illustration of deformations for parallel and perpendicular crystallites in P(NDI2OD-T2) thin film during tensile stretching: (a) chain-slippage for parallel crystallites; (b) chain-peeling for perpendicular crystallites.

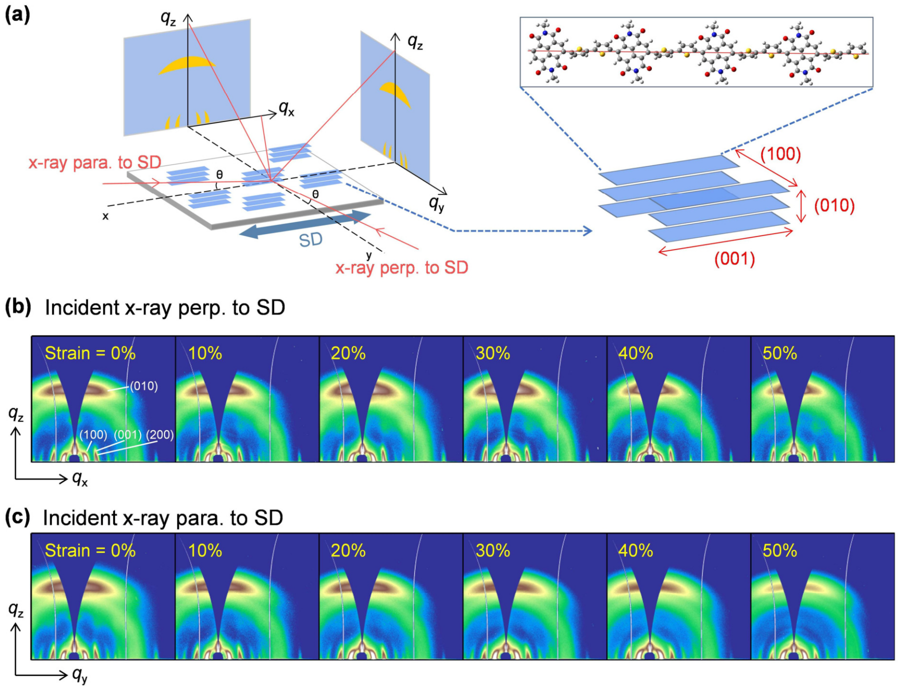

Supplementary Figure 28. (a) Schematic representation of GIWAXS geometries for strained P(NDI2OD-T2) thin film, where the scattering images are collected with the incident X-ray roughly perpendicular (perp.) or parallel (para.) to the SD. (b) GIWAXS patterns of P(NDI2OD-T2) thin films with various strains measured with the incident X-ray beam (b) perpendicular (q_x) and (c) parallel (q_y) to the SD.

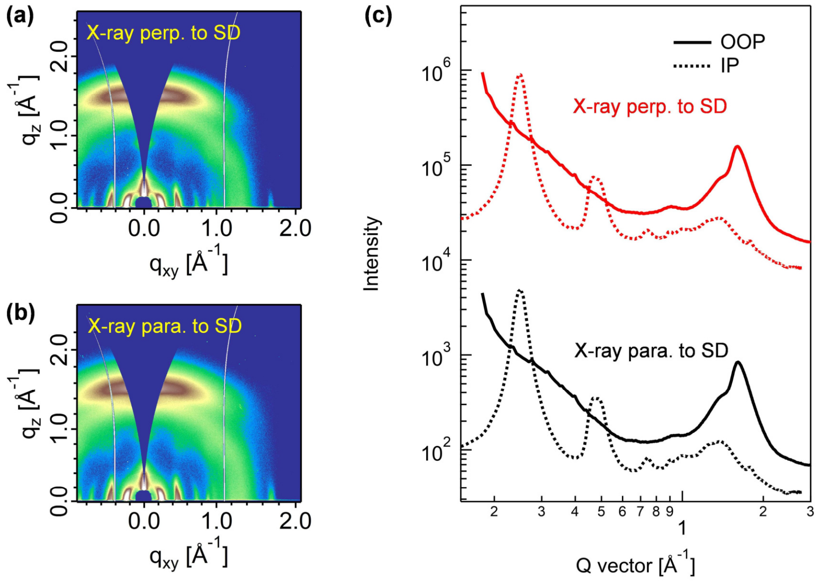

Supplementary Figure 29. GIWAXS (a,b) 2D images and (c) averaged *I*-*q* curves in IP and OOP directions of P(NDI2OD-T2) thin film under pre-stretched condition.

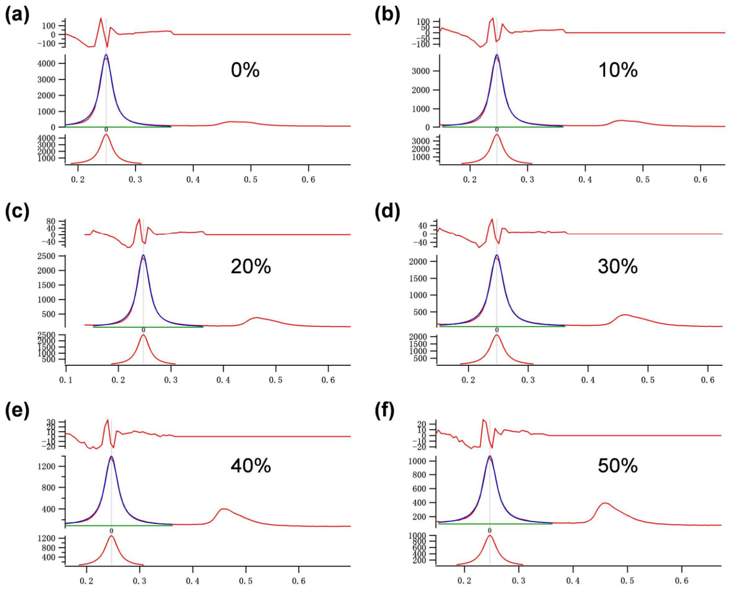

Supplementary Figure 30. Fitting illustration of GIWAXS (100) reflections in the q_x direction (X-ray beam perpendicular to the stretch direction) of P(NDI2OD-T2) thin films under different strains: (a) 0%, (b) 10%, (c) 20%, (d) 30%, (e) 40%, and (f) 50%.

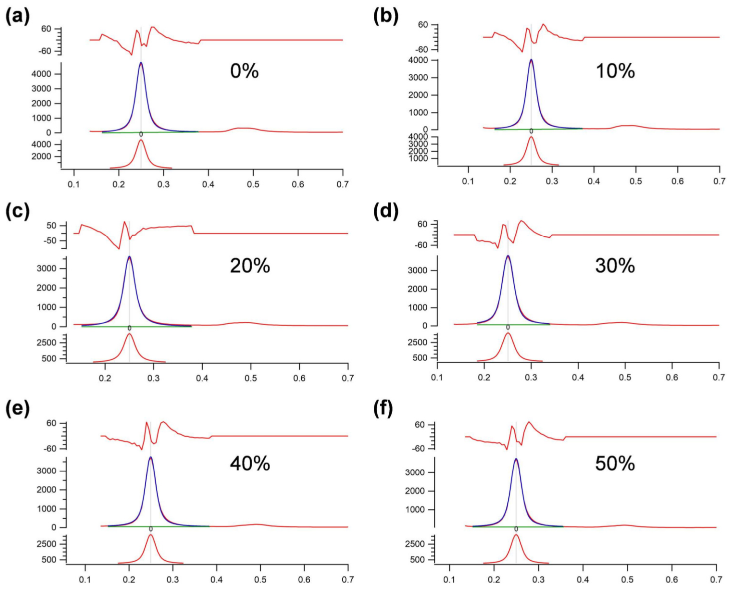

Supplementary Figure 31. Fitting illustration of GIWAXS (100) reflections in the q_y direction (X-ray beam parallel to the stretch direction) of P(NDI2OD-T2) thin films under different strains: (a) 0%, (b) 10%, (c) 20%, (d) 30%, (e) 40%, and (f) 50%.

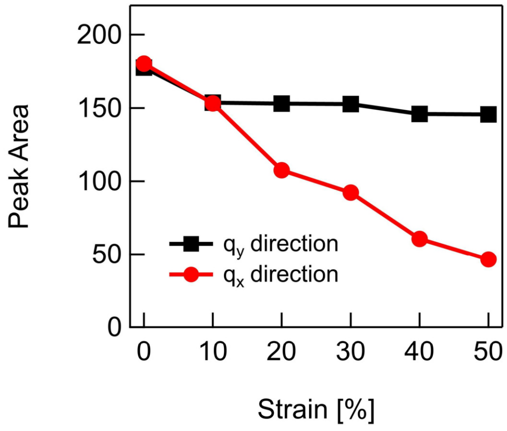

Supplementary Figure 32. GIWAXS (100) peak area in the q_x and q_y directions as a function of strain for P(NDI2OD-T2) thin films

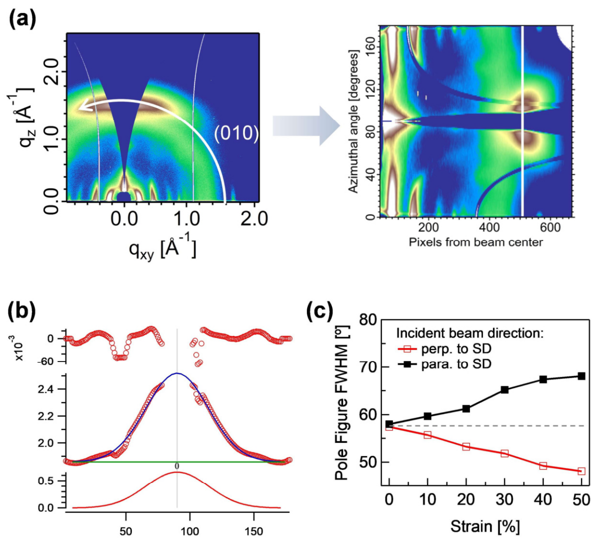

Supplementary Figure 33. (a) Illustration of the circular average for the generation of (010) pole figures and (b) fitting example for the pole figures using Gaussian function. (c) (010) pole figure full width at half maximum (FWHM) as functions of strain.

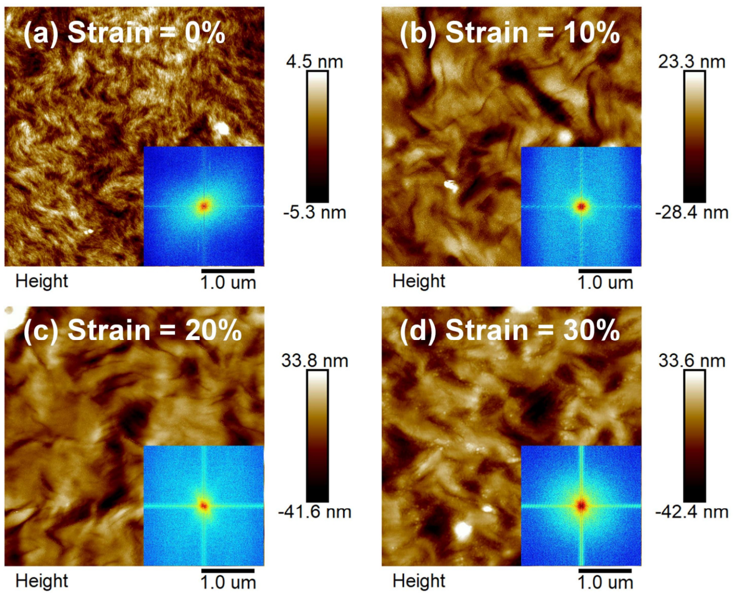

Supplementary Figure 34. AFM height images and corresponding FFT images of P(NDI2OD-T2) thin films under different stretch strains: (a) 0%, (b) 10%, (c) 20%, and (d) 30%. The stretch direction is in the horizontal direction of the images.

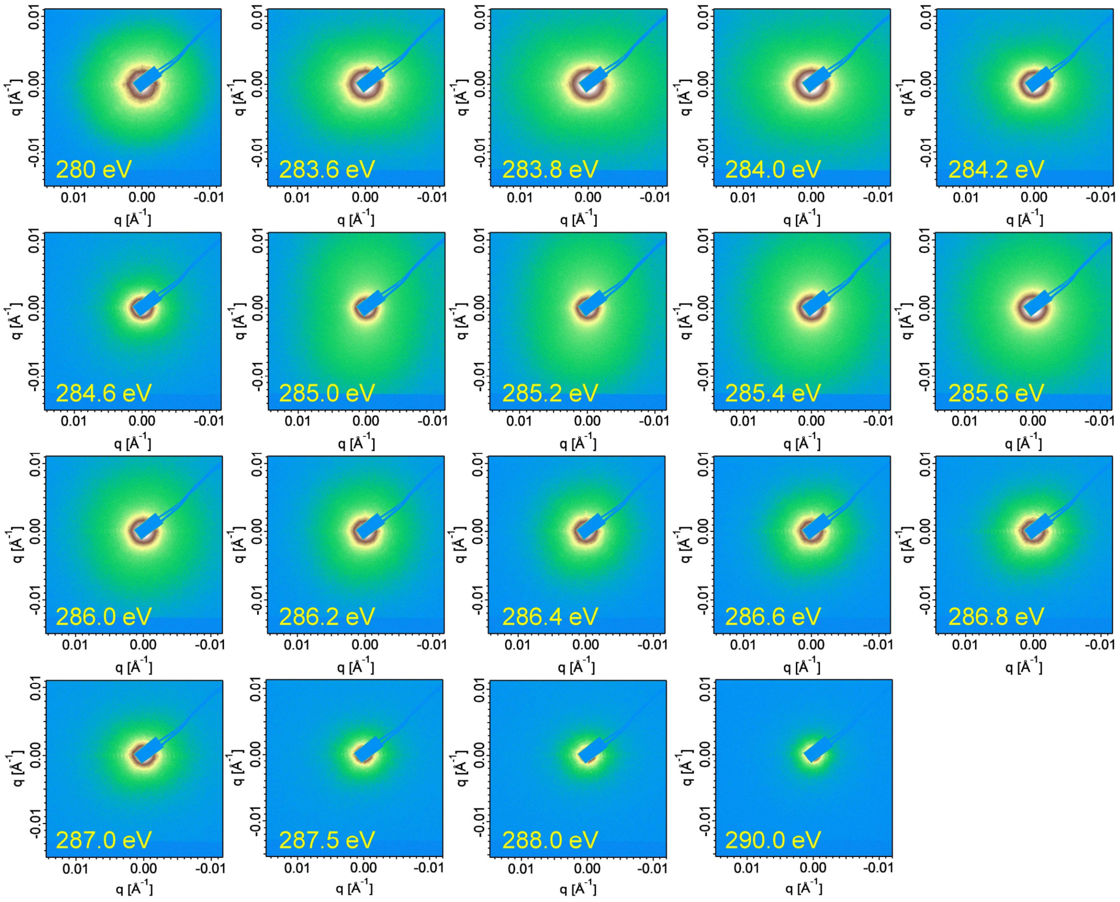

Supplementary Figure 35. RSoXS patterns of a pre-stretched P(NDI2OD-T2) thin film recorded under various incident X-ray energies. The X-ray had a horizontal polarization.

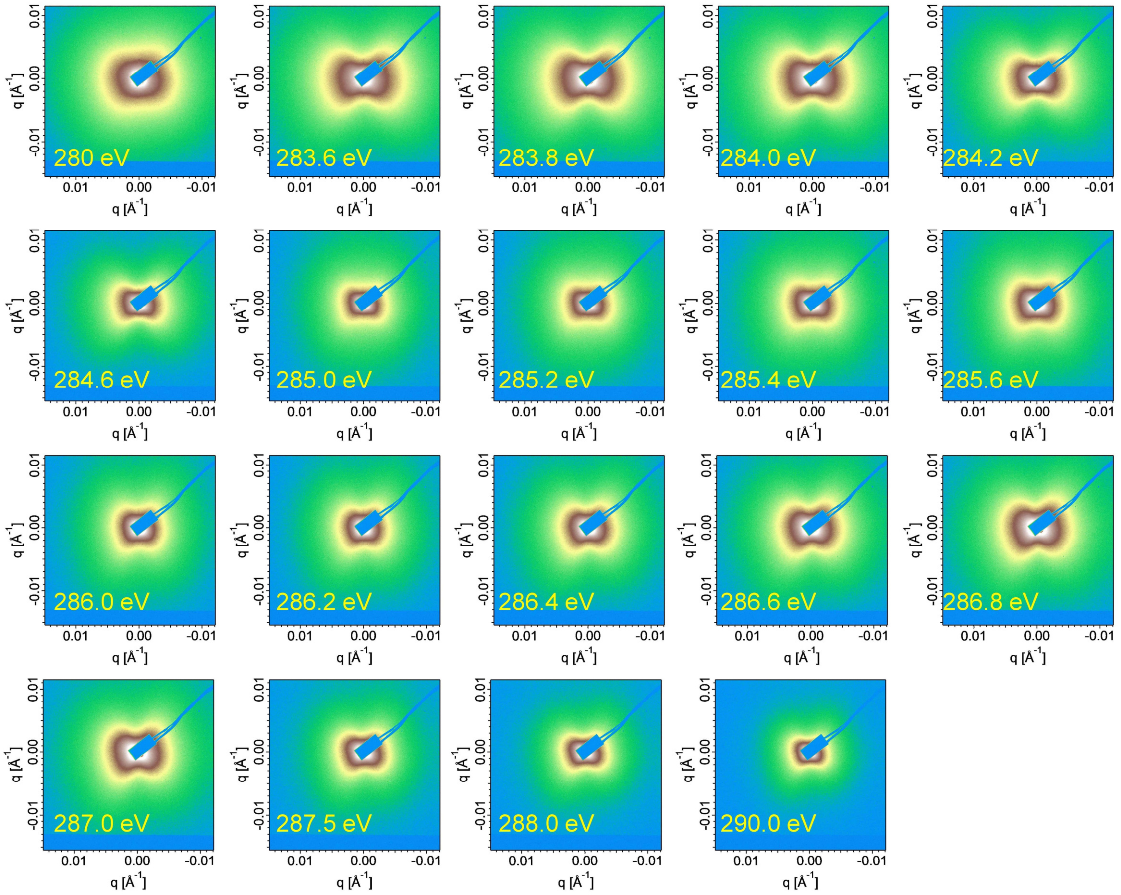

Supplementary Figure 36. RSoXS patterns of a 30%-strained P(NDI2OD-T2) thin film recorded under various incident X-ray energies. The X-ray had a horizontal polarization.

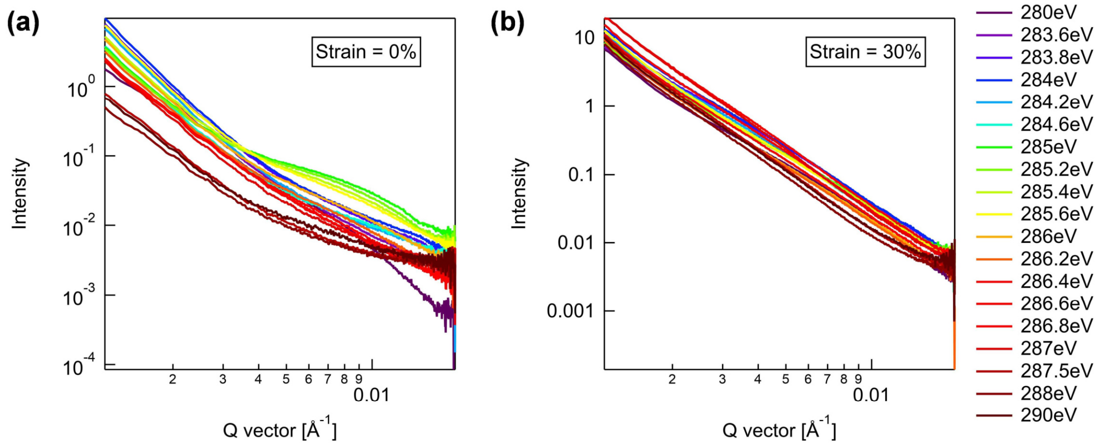

Supplementary Figure 37. RSoXS *I*-*q* curves of P(NDI2OD-T2) thin films under (a) 0% and (b) 30% strain.

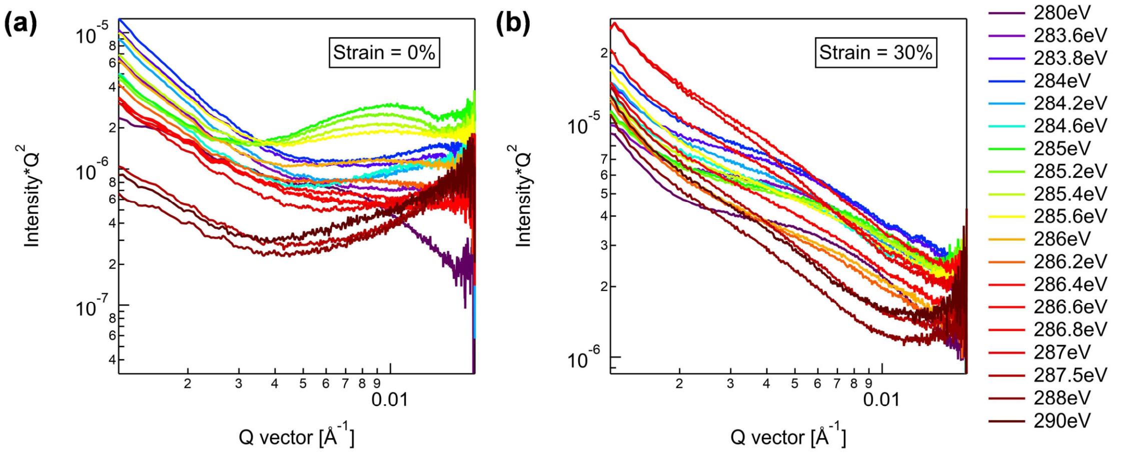

Supplementary Figure 38. RSoXS Iq^2-q curves of P(NDI2OD-T2) thin films under (a) 0% and (b) 30% strain.

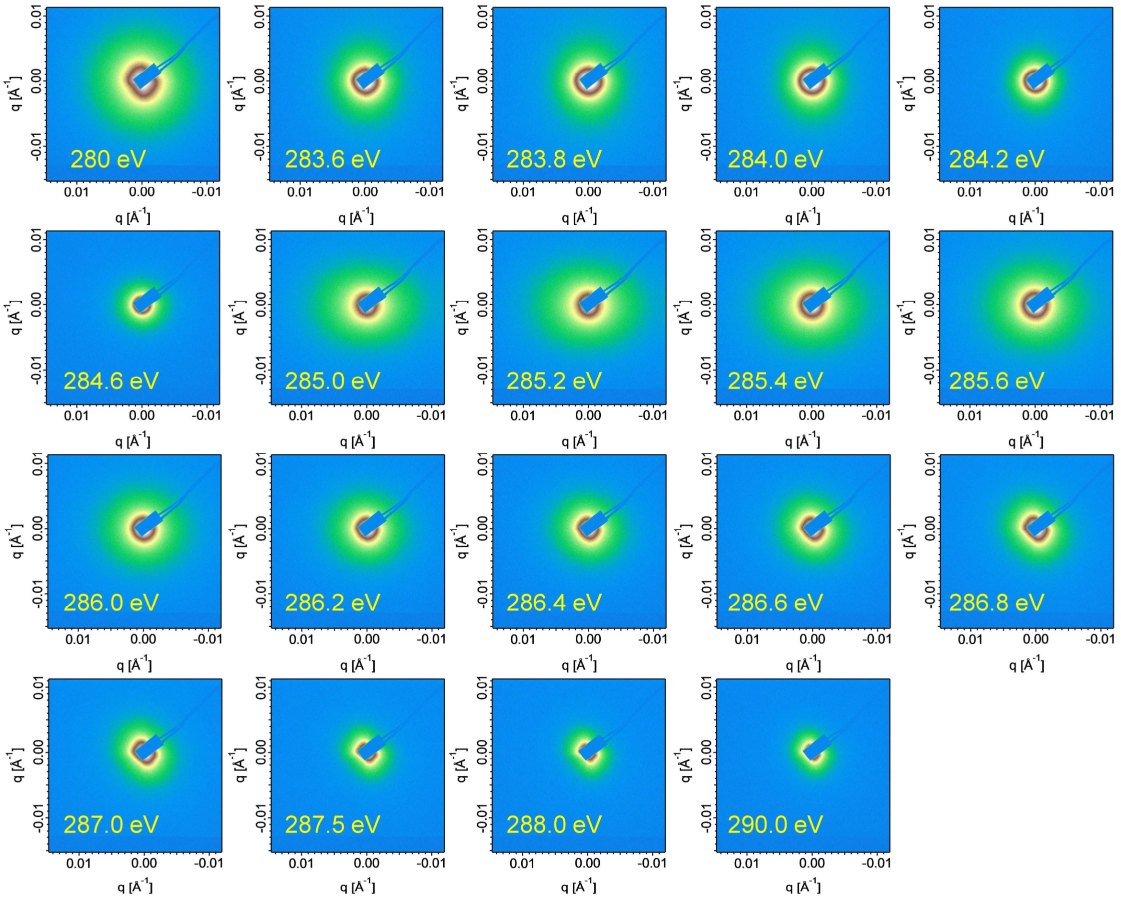

Supplementary Figure 39. RSoXS patterns of a pre-stretched P(NDI2OD-T2) thin film recorded under various incident X-ray energies. The X-ray had a vertical polarization.

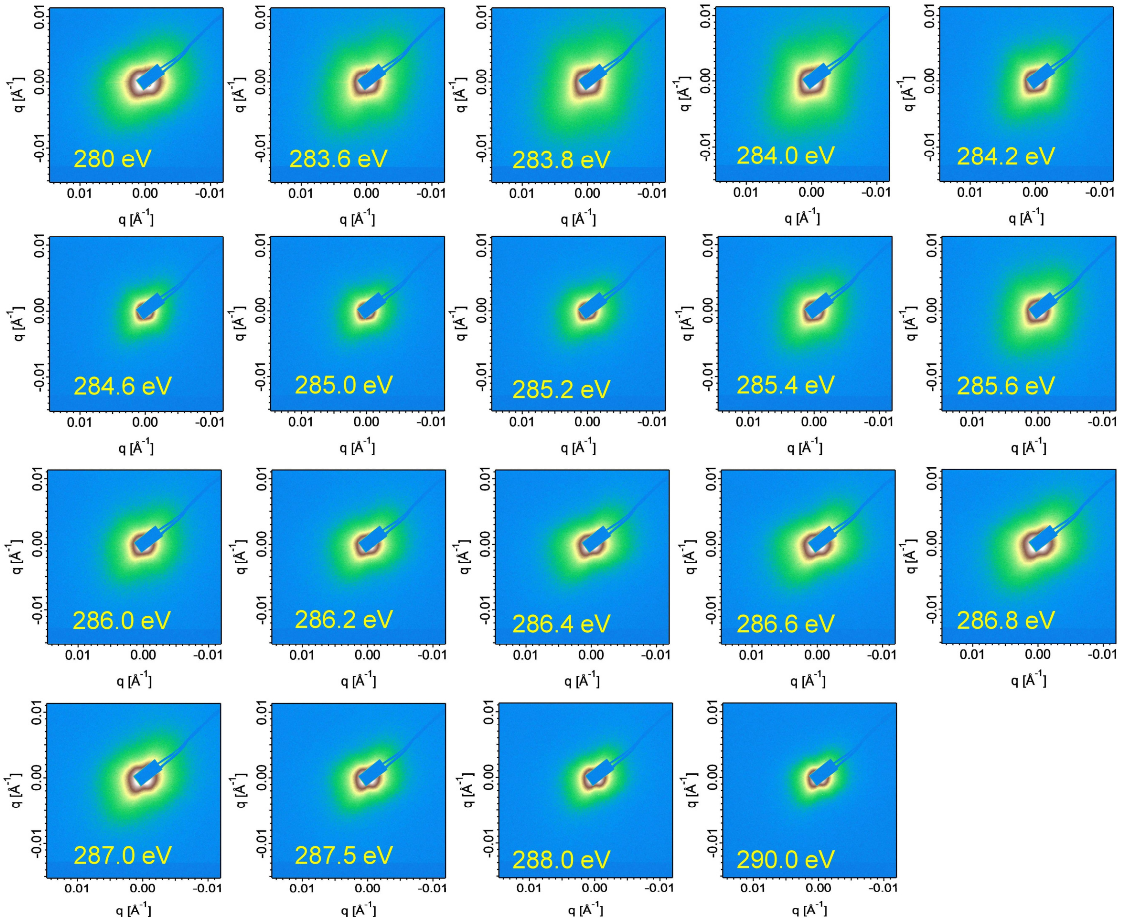

Supplementary Figure 40. RSoXS patterns of a 30%-strained P(NDI2OD-T2) thin film recorded under various incident X-ray energies. The X-ray had a vertical polarization.

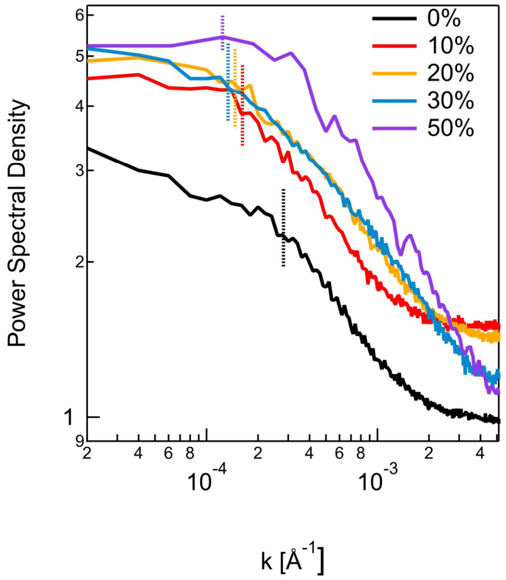

Supplementary Figure 41. Power spectral density (PSD) plots extracted from AFM height images (as shown in Supplementary Figure 34 for 0-30% strains and Fig. 3a for 50% strain) of P(NDI2OD-T2) thin films under different stretch strains. The dotted lines highlight the turning or peak positions used to estimate the characteristic domain size, calculated as the inverse of the corresponding spatial frequency *k*. The characteristic domain size increases from 3.6 μm to 6.2 μm, 7.1 μm, 7.7 μm, and 8.3 μm for films strained at 0%, 10%, 20%, 30%, and 50%, respectively. These results reflect the micrometer-scale aggregation process captured by AFM.

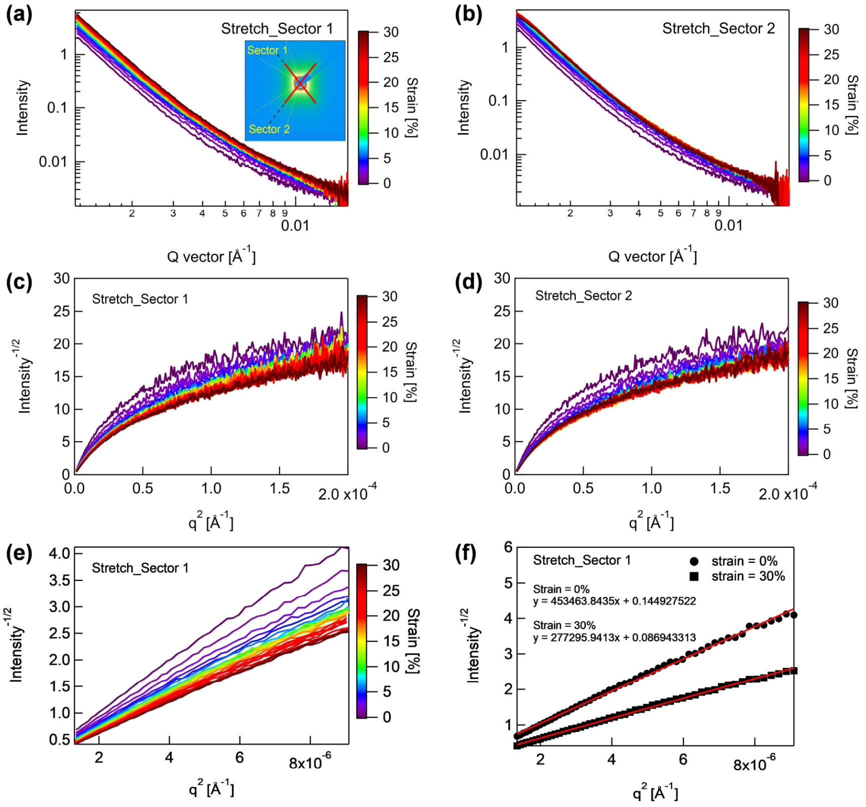

Supplementary Figure 42. (a-b) The *I*-*q* curves, averaged at two azimuthal angles of the X-shaped signal in RSoXS, i.e., φ_1 (Sector 1) and φ_2 (Sector 2). Inset shows the sector averaging details of Sector 1 and Sector 2. (c-d) The $I^{-1/2}$-q^2 curves plotted following the Debye-Beuche model, $(I(q))^{-1/2} = K(a^3Q)^{-1/2}(1 + a^2q^2)$, where K is a constant, Q is the scattering invariant, and a is the correlation length, which described the average domain size. The correlation lengths were fitted from the linear region at low-q range (0.0012-0.003 $Å^{-1}$). (e) Representative fit region (0.001-0.003 $Å^{-1}$) of the $I^{-1/2}$-q^2 curves. (f) Representative linear fits of the $I^{-1/2}$-q^2 curves, where the resultant correlation lengths equal to the values of $(\text{slope/intercept})^{-1/2}$ [Å].

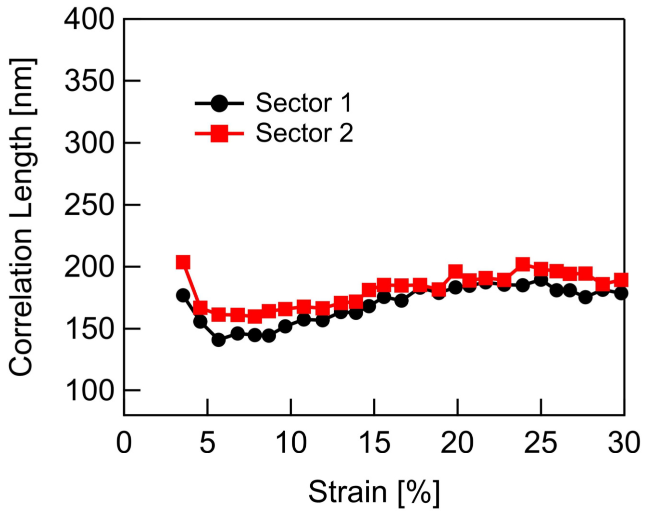

Supplementary Figure 43. Correlation length as a function of film strain of the P(NDI2OD-T2) thin film. The correlation length in both section 1 and section 2 first decreased by ~50 nm during the initial strain (<5%), likely due to transverse compression, and subsequently increased from ~150 nm to ~185 nm as strain progressed. This trend complements the micro-scale aggregation observed by AFM (Supplementary Figure 41).

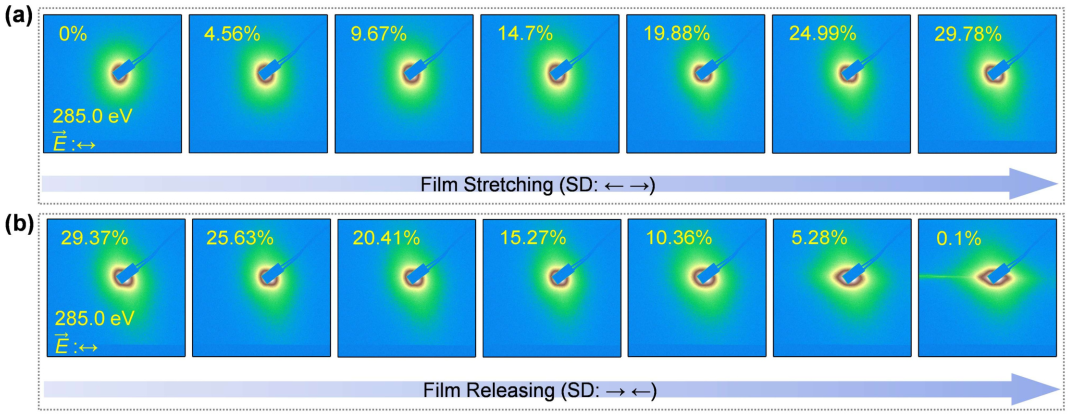

Supplementary Figure 44. In situ RSoXS images of a P(NDI2OD-T2) thin film at different strains during (a) stretching and (b) releasing, which are probed at 285.0 eV.

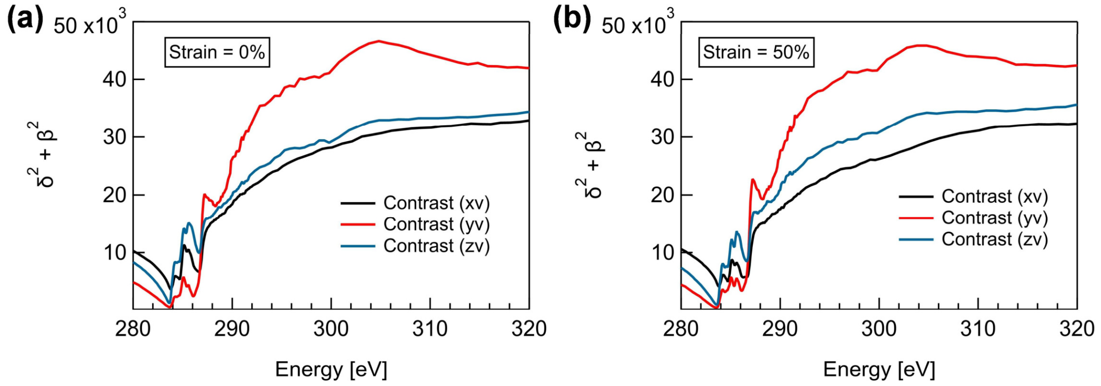

Supplementary Figure 45. Vacuum/roughness contrast in x, y, and z direction of the P(NDI2OD-T2) thin film under (a) 0% and (b) 50% strain.

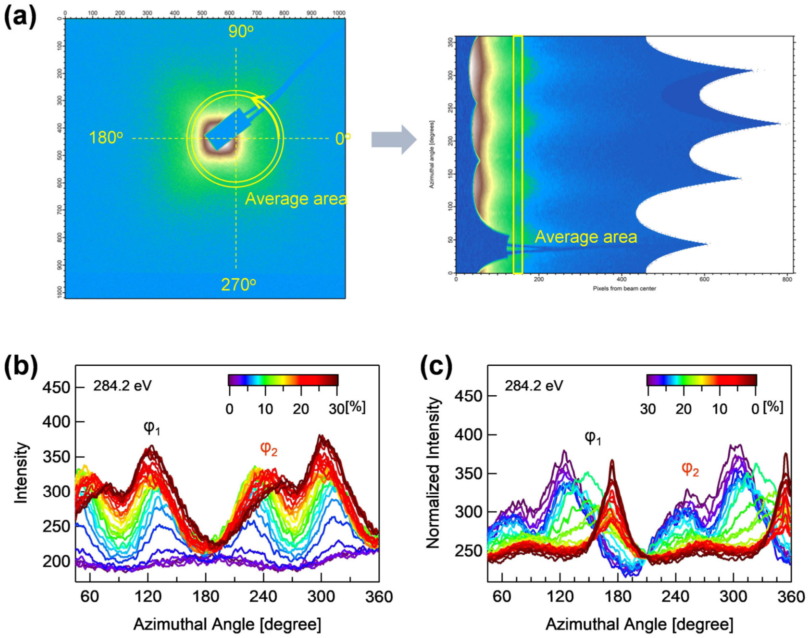

Supplementary Figure 46. (a) Illustration of the circular average area for the extraction of the azimuthal intensities from RSoXS images. Azimuthal averaging means to take the average of the scattering intensity along a circular path centered at the beam center (or q = 0). The result of azimuthal averaging is a 1D curves, which shows the scattering intensity as a function of azimuthal angle. The azimuthal intensities were averaged at a q value of 0.004 $Å^{-1}$ with a Δq width of 0.001 $Å^{-1}$. Azimuthal intensities extracted from the patterns at 284.0 eV of a P(NDI2OD-T2) thin film during a stretching-releasing cycle: (b) stretching process; (c) releasing process. The azimuthal intensities of releasing process are normalized at 210° for clarity, where the intensities remain relatively low in this process.

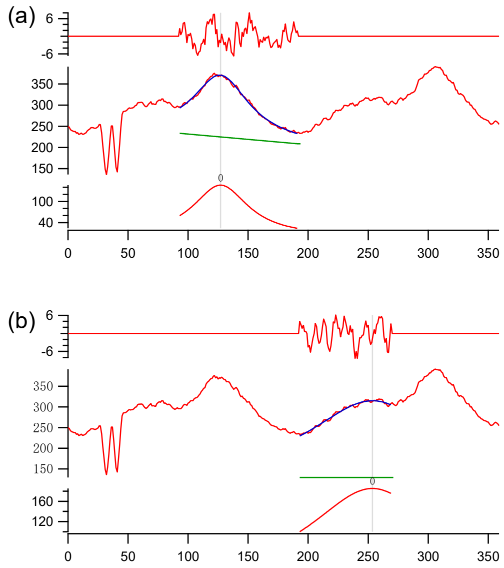

Supplementary Figure 47. Example of Gaussian-based peak fitting used to extract (a) φ_1 and (b) φ_2 from azimuthal intensity profiles obtained at 284.0 eV.

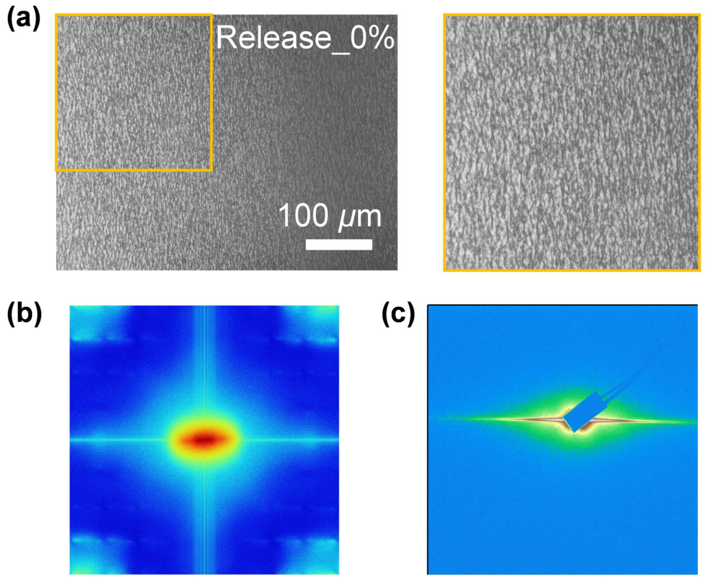

Supplementary Figure 48. (a) Optical microscopy image of the P(NDI2OD-T2) thin film which was stretched to 50% strain and then released to 0%. (b) FFT pattern of the optical microscopy image. (c) Experimental RSoXS image of the P(NDI2OD-T2) thin film which was stretched to 50% strain and then released to 0%.

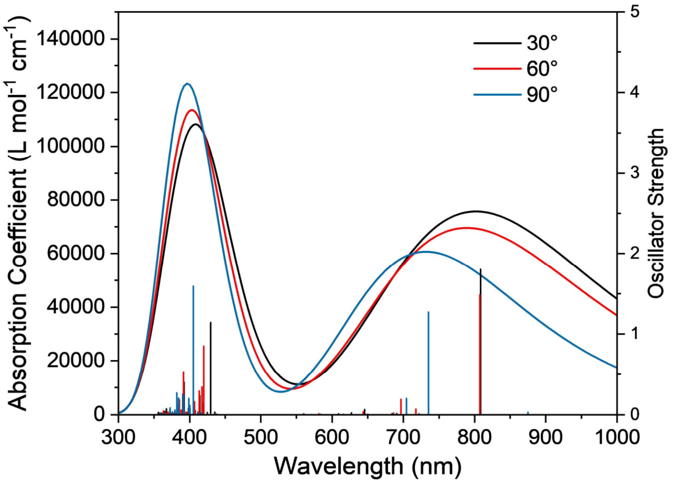

Supplementary Figure 49. Time-dependent density functional theory (TD-DFT) calculated absorption spectra of strain-dependent dimer structures with different the dihedral angle between BT and NDI units.

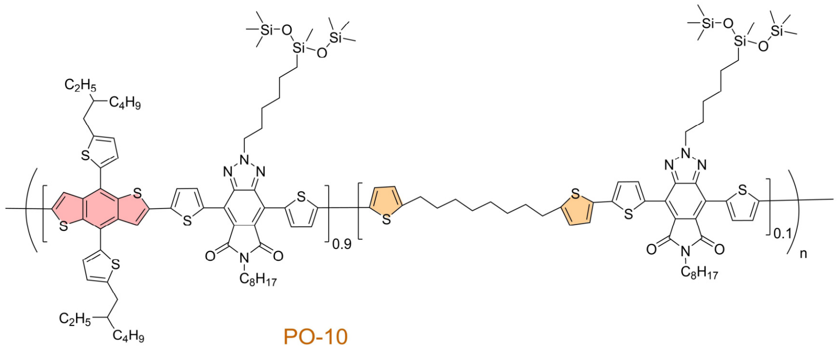

Supplementary Figure 50. Chemical structure of PO10.

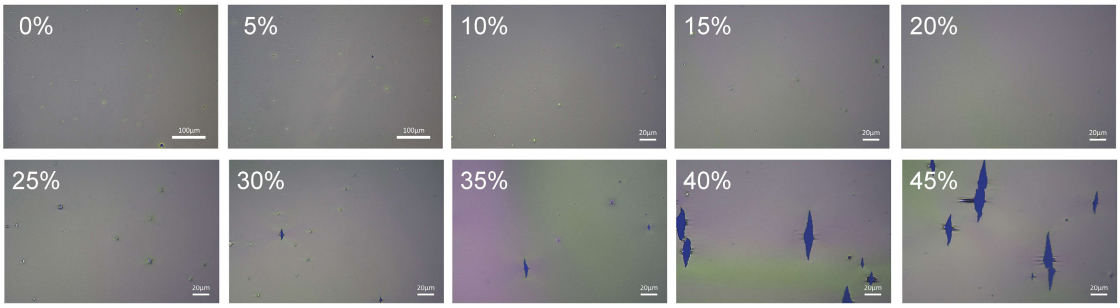

Supplementary Figure 51. Optical microscopy images of PO10:P(NDI2OD-T2) (2:1, wt:wt) blend thin film at different strains. Small microcracks appeared when the films were stretched to 30% strain, representing a crack onset strain (COS) of 30%.

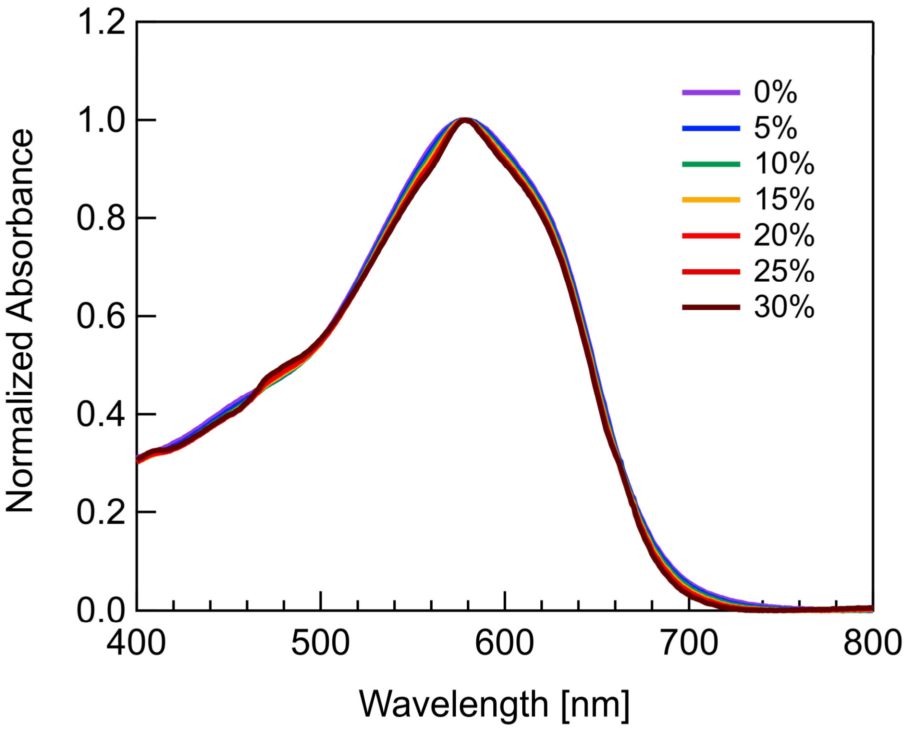

Supplementary Figure 52. Normalized absorption spectra of the neat PO10 thin film under different stretch strains with a film-on-elastomer sample.

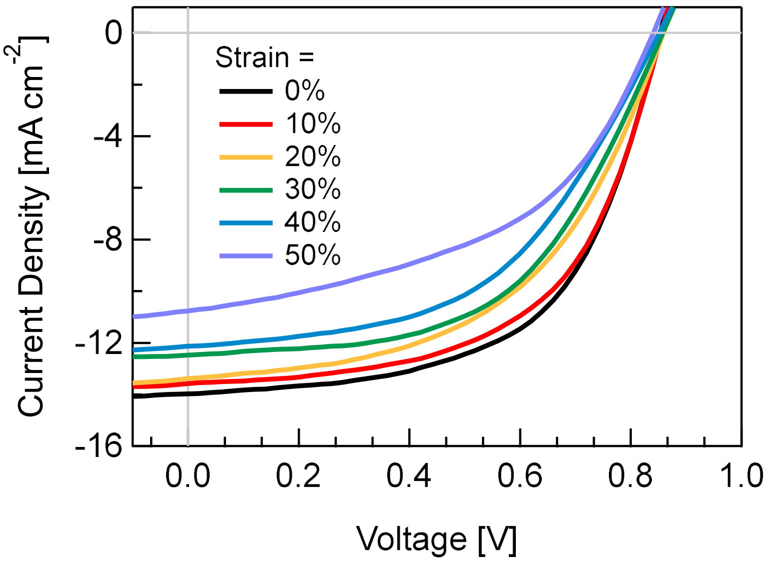

Supplementary Figure 53. *J-V* curves of PO10:P(NDI2OD-T2) (2:1, wt:wt) based intrinsically stretchable OPV devices at varying strains.

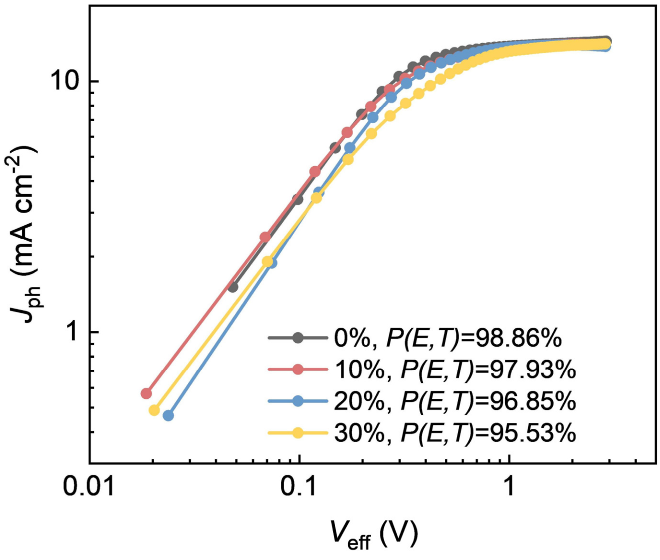

Supplementary Figure 54. J_{ph}-V_{eff} curves of PO10:P(NDI2OD-T2) based stretchable OPV device under different strains.

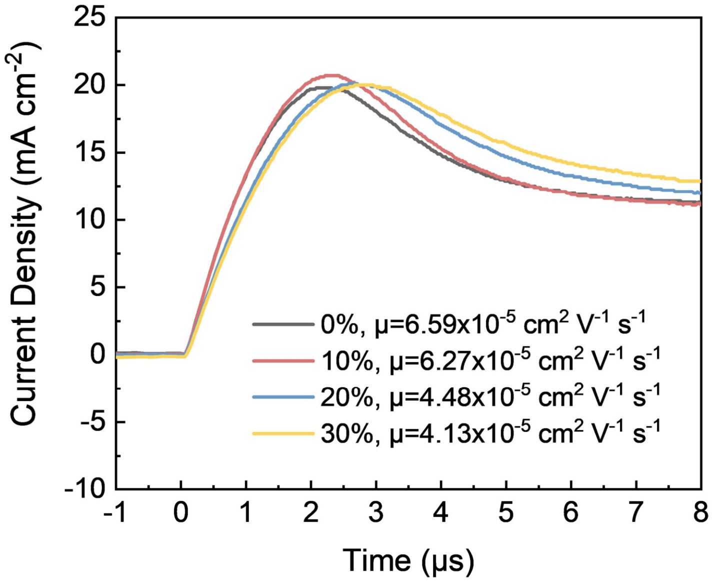

Supplementary Figure 55. Photo-CELIV curves and the corresponding mobilities (μ) of PO10:P(NDI2OD-T2) based stretchable OPV device under different strains.

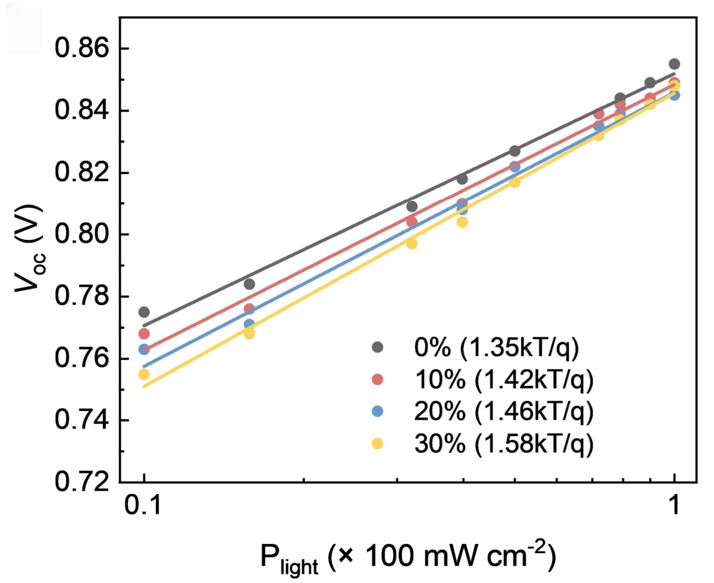

Supplementary Figure 56. Light-intensity (P_{light}) dependency of V_{OC} of PO10:P(NDI2OD-T2) based stretchable OPV device under different strains.

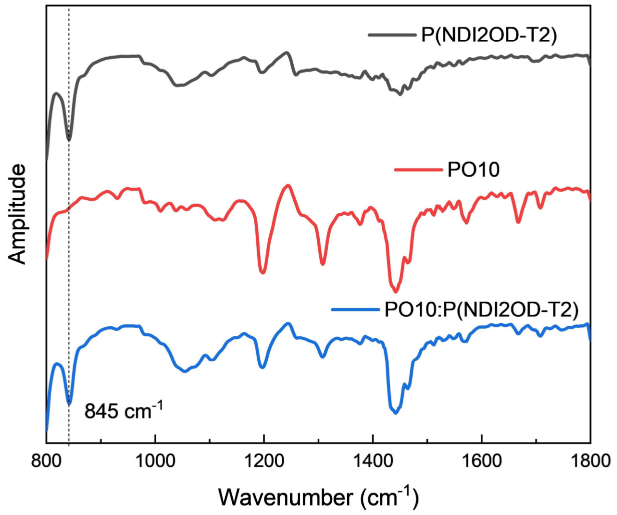

Supplementary Figure 57. IR spectra of neat PO10, P(NDI2OD-T2), and PO10:P(NDI2OD-T2) (2:1) blend film, which indicate that P(NDI2OD-T2) domains in the blend film can be highlighted by the characteristic wavenumber of 845 cm^{-1}.

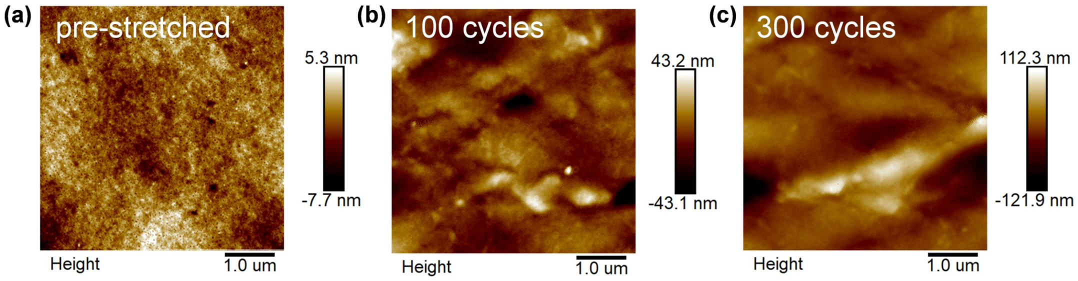

Supplementary Figure 58. AFM height images of PO10:P(NDI2OD-T2) thin films on PDMS substrates under (a) pre-stretched condition, (b) after 100 cycles at 20% strain, and (c) after 300 cycles at 20% strain.

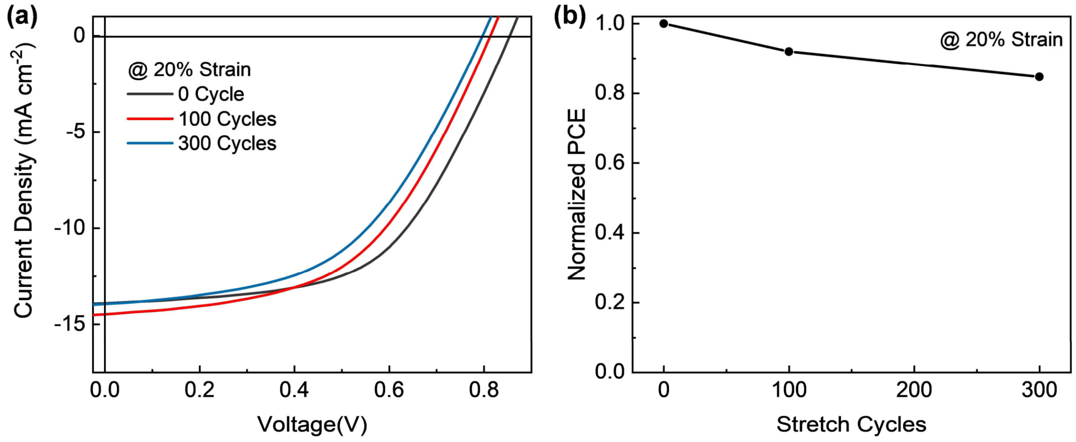

Supplementary Figure 59. (a) *J-V* curves and (b) normalized PCE of stretchable OPV device based on PO10:P(NDI2OD-T2) thin films under pre-stretched condition, after 100 cycles at 20% strain, and after 300 cycles at 20% strain.

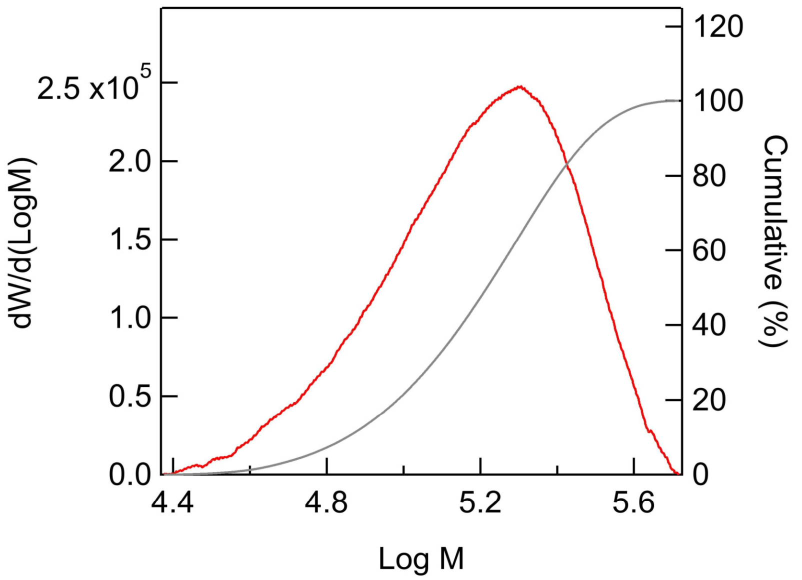

Supplementary Figure 60. Molecular weight distribution and cumulative curves of P(NDI2OD-T2).

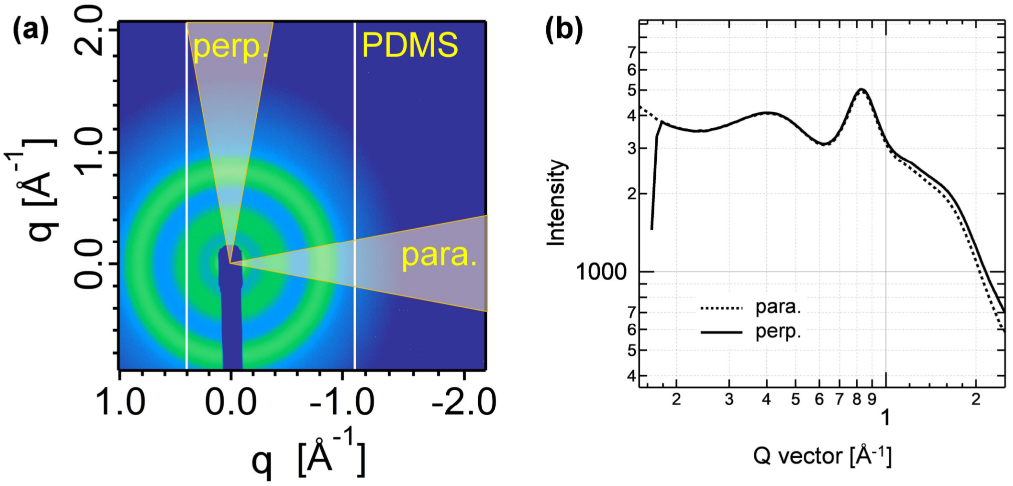

Supplementary Figure 61. WAXS (a) 2D image and (b) *I*-*q* curves averaged in the parallel (para.) and perpendicular (perp.) directions.

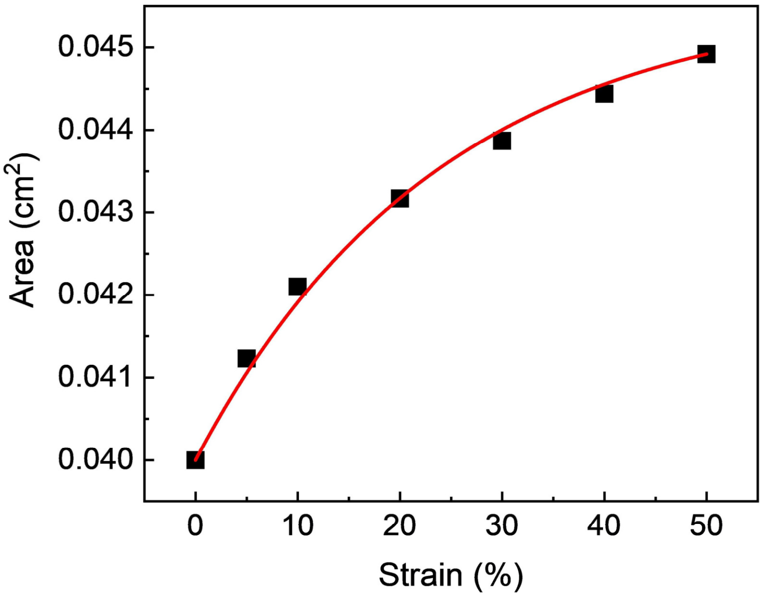

Supplementary Figure 62. The correction curve for the active area of intrinsically stretchable OPV devices. Black squares denote the averaged active areas of three independent devices, while the red line represents the corresponding fit using a three-phase exponential decay function.

Supplementary Tables

Supplementary Table 1. Fitted results of the GIWAXS (100) reflections in the q_x direction of P(NDI2OD-T2) thin film under different strains. FWHM represents full width at half maximum.

Strain [%]	Type	Location [$Å^{-1}$]	Amplitude	Area	FWHM [$Å^{-1}$]
0	Lorentzian	0.25	4545.02	180.37	0.0253
10	Lorentzian	0.25	3823.81	153.09	0.0255
20	Lorentzian	0.25	2492.22	107.47	0.0275
30	Lorentzian	0.25	2127.76	92.28	0.0276
40	Lorentzian	0.25	1299.00	60.51	0.0297
50	Lorentzian	0.25	982.19	46.52	0.0302

Supplementary Table 2. Fitted results of the GIWAXS (100) reflections in the q_y direction of P(NDI2OD-T2) thin film under different strains. FWHM represents full width at half maximum.

Strain [%]	Type	Location [$Å^{-1}$]	Amplitude	Area	FWHM [$Å^{-1}$]
0	Lorentzian	0.25	4769.91	177.63	0.0260
10	Lorentzian	0.25	4014.66	153.62	0.0267
20	Lorentzian	0.25	3737.5	152.97	0.0296
30	Lorentzian	0.25	3643.62	152.61	0.0280
40	Lorentzian	0.25	3700.64	145.93	0.0285
50	Lorentzian	0.25	3705.69	145.55	0.0280

Supplementary Table 3. Photovoltaic parameters of PO10:P(NDI2OD-T2) (2:1, wt:wt) based intrinsically stretchable OPV devices at varying strains.

Strain [%]	V_{OC} [V]	J_{SC} [mA cm^{-2}]	FF [%]	PCE [%]
0	0.854	13.97	58.00	6.92
10	0.856	13.58	56.82	6.56
20	0.859	13.39	51.28	5.96
30	0.858	12.48	53.97	5.78
40	0.852	12.14	50.33	5.21
50	0.841	10.76	47.63	4.31

Supplementary Table 4. Device parameters of stretchable OPV device based on PO10:P(NDI2OD-T2) thin films under cyclic stretch at 20% strain.

Cycles	V_{OC} [V]	J_{SC} [mA cm^{-2}]	FF [%]	PCE [%]	Normalized PCE
0	0.855	13.91	55.67	6.62	1
100	0.813	14.47	51.73	6.09	0.92
300	0.797	13.94	50.50	5.61	0.85
