# Supplementary material for: Correlative molecular-to-mesoscale evolution in conjugated polymers for intrinsically stretchable organic photovoltaics
Source: Nat Commun. 2026 Feb 20;17:2980. doi: 10.1038/s41467-025-68265-4 (PMC13035893; doi:10.1038/s41467-025-68265-4)
Supplement: Supplementary file 2 — Reporting Summary [file 41467_2025_68265_MOESM2_ESM.pdf]

nature portfolio

Corresponding author(s): Wenkai Zhong, Cheng Wang, Feng Liu

Solar Cells Reporting Summary

Nature Portfolio wishes to improve the reproducibility of the work that we publish. This form is intended for publication with all accepted papers reporting the characterization of photovoltaic devices and provides structure for consistency and transparency in reporting. Some list items might not apply to an individual manuscript, but all fields must be completed for clarity.

For further information on Nature Research policies, including our data availability policy, see Authors & Referees.

• Experimental design

Please check the following details are reported in the manuscript, and provide a brief description or explanation where applicable.

1. Dimensions

Area of the tested solar cells

- [x] Yes — The initial active area of the stretchable solar cells was 0.04 cm^2.
- [ ] No — *Explain why this information is not reported/not relevant.*

Method used to determine the device area

- [x] Yes — The initial device area (0.04 cm^2) was defined by the overlap of a patterned M-PH1000 bottom electrode and a patterned EGaIn@Ag top electrode, forming a cross-electrode geometry. During mechanical stretching, the active area was altered due to geometric deformation. To account for this, we applied a correction factor to the J–V curves. The corrected active area was calculated by averaging area changes from three independent devices under the same strain conditions. The dimensional changes were measured using optical microscopy.
- [ ] No — *Explain why this information is not reported/not relevant.*

2. Current-voltage characterization

Current density-voltage (J-V) plots in both forward and backward direction

- [ ] Yes — J–V measurements were performed in a single forward scan direction from –0.2 V to 1.0 V. No backward scan was conducted, as the devices exhibited negligible hysteresis in preliminary testing.
- [x] No

Voltage scan conditions

- [x] Yes — J–V scans were recorded using a Keithley 2400 under AM 1.5G (100 mW cm^{-2}) illumination, with a delay time of 1 ms.
- [ ] No — *Explain why this information is not reported/not relevant.*

Test environment

- [x] Yes — All measurements were performed inside a nitrogen-filled glovebox with O_2 and H_2O levels below 1 ppm.
- [ ] No — *Explain why this information is not reported/not relevant.*

Protocol for preconditioning of the device before its characterization

- [x] Yes — Devices were stabilized under 1-sun illumination for 10 seconds before J–V measurements.
- [ ] No — *Explain why this information is not reported/not relevant.*

Stability of the J-V characteristic

- [ ] Yes — *Provide a description of the method used. The stability of the J-V characteristic can be verified with time evolution of the maximum power point or with the photocurrent at maximum power point; see ref. 5 for details.*
- [x] No — The main focus of this study was to investigate the strain-induced structural changes and the influence on mechanical properties and photovoltaic performance of intrinsically stretchable OPVs. While device performance was repeatable under static conditions, long-term stability under bias was not within the scope of this work.

3. Hysteresis or any other unusual behaviour

Description of the unusual behaviour observed during the characterization

- [ ] Yes — *Provide a description of hysteresis or any other unusual behaviour observed during the characterization.*
- [x] No — Only forward J–V scans (from –0.2 V to 1.0 V) were performed.

Related experimental data

- [ ] Yes — *Provide a description of the related experimental data.*
- [x] No — Not applicable.

4. Efficiency

External quantum efficiency (EQE) or incident photons to current efficiency (IPCE)	☐ Yes<br>☒ No	*Provide a description of the technique used.*<br>EQE measurements were not performed due to tensile stage limitations in the EQE setup, which was not compatible with the stretchable device structure and geometry.
A comparison between the integrated response under the standard reference spectrum and the response measure under the simulator	☐ Yes<br>☒ No	*State where this information can be found in the text.*<br>Not applicable.
For tandem solar cells, the bias illumination and bias voltage used for each subcell	☐ Yes<br>☒ No	*Provide a description of the measurement conditions.*<br>Not applicable.

5. Calibration

Light source and reference cell or sensor used for the characterization	☒ Yes<br>☐ No	A SS-F5-3A solar simulator (Enlitech) calibrated with a certified silicon reference cell (SRC-2020) was used.<br>*Explain why this information is not reported/not relevant.*
Confirmation that the reference cell was calibrated and certified	☒ Yes<br>☐ No	The reference cell was calibrated and traceable to NIST.<br>*Explain why this information is not reported/not relevant.*
Calculation of spectral mismatch between the reference cell and the devices under test	☐ Yes<br>☒ No	*Provide a value of the spectral mismatch and/or a description of how it has been taken into account in the measurements.*<br>Not applicable.

6. Mask/aperture

Size of the mask/aperture used during testing	☐ Yes<br>☒ No	*Report the size of the mask/aperture.*<br>Not applicable.
Variation of the measured short-circuit current density with the mask/aperture area	☐ Yes<br>☒ No	*Report the difference in the short-circuit current density values measured with the mask and aperture area.*<br>Not applicable.

7. Performance certification

Identity of the independent certification laboratory that confirmed the photovoltaic performance	☐ Yes<br>☒ No	*Identify the independent certification laboratory.*<br>Not applicable.
A copy of any certificate(s)	☐ Yes<br>☒ No	*Certificate copies should be provided in the Supplementary information. Please state the supplementary item number.*<br>Not applicable.

8. Statistics

Number of solar cells tested	☐ Yes<br>☒ No	*Report how many solar cells have been tested, specifying the number of individual substrates.*<br>Not applicable.
Statistical analysis of the device performance	☐ Yes<br>☒ No	*State where this information can be found in the text.*<br>Not applicable.

9. Long-term stability analysis

Type of analysis, bias conditions and environmental conditions	☐ Yes<br>☒ No	*Provide a description of the type of analysis, bias conditions and environmental conditions (e.g. illumination type, temperature, atmosphere humidity, encapsulation method, preconditioning temperature, bias) for each long-term stability analysis carried out; see ref. 7 and 8 for details.*<br>Not applicable.
